# Strategies to achieve a carbon neutral society: a review

**DOI:** 10.1007/s10311-022-01435-8

**Published:** 2022-04-08

**Authors:** Lin Chen, Goodluck Msigwa, Mingyu Yang, Ahmed I. Osman, Samer Fawzy, David W. Rooney, Pow-Seng Yap

**Affiliations:** 1 Department of Civil Engineering, Xi’an Jiaotong-Liverpool University, Suzhou, 215123 China; 2 School of Chemistry and Chemical Engineering, David Keir Building, Queen’s University Belfast, Stranmillis Road, Northern Ireland, Belfast, BT9 5AG UK

**Keywords:** Carbon neutrality, Net-zero carbon plan, Worldwide initiatives, Carbon emissions, Carbon neutral system, Life cycle analysis

## Abstract

The increasing global industrialization and over-exploitation of fossil fuels has induced the release of greenhouse gases, leading to an increase in global temperature and causing environmental issues. There is therefore an urgent necessity to reach net-zero carbon emissions. Only 4.5% of countries have achieved carbon neutrality, and most countries are still planning to do so by 2050–2070. Moreover, synergies between different countries have hampered synergies between adaptation and mitigation policies, as well as their co-benefits. Here, we present a strategy to reach a carbon neutral economy by examining the outcome goals of the 26th summit of the United Nations Climate Change Conference of the Parties (COP 26). Methods have been designed for mapping carbon emissions, such as input–output models, spatial systems, geographic information system maps, light detection and ranging techniques, and logarithmic mean divisia. We present decarbonization technologies and initiatives, and negative emissions technologies, and we discuss carbon trading and carbon tax. We propose plans for carbon neutrality such as shifting away from fossil fuels toward renewable energy, and the development of low-carbon technologies, low-carbon agriculture, changing dietary habits and increasing the value of food and agricultural waste. Developing resilient buildings and cities, introducing decentralized energy systems, and the electrification of the transportation sector is also necessary. We also review the life cycle analysis of carbon neutral systems.

## Introduction

With the increasing global industrialization and over-exploitation of non-renewable energy sources, a large number of greenhouse gases have been released, leading to an increase in global temperature and causing a series of environmental degradation issues (Wang et al. 2021). From pre-industrialization, around 1850, until 2022, the global average atmospheric carbon dioxide (CO_2_) concentration increased substantially from 285 to 419 ppm (Chen 2021; CO_2_ Daily 2022). As a result, the United Kingdom meteorological office estimates a global average surface temperature increase of about 0.97 to 1.21 °C throughout 1850–2022, with a central estimate of 1.09 °C; they also predict that 2022 will continue the trend of the warmest years in the world (Sangomla 2022). Furthermore, global greenhouse gas emissions are expected to rise by 50% by 2050, owing primarily to CO_2_ emissions from non-renewable energy use (Rabaey and Ragauskas 2014). Without effective measures or technologies to reduce or control CO_2_ emissions, the global average atmospheric CO_2_ concentration, as well as the global surface and ocean temperatures, will continue to rise. The rising global temperature caused by these greenhouse gases has already caused significant damage to the human living environment, including the extinction of some species, loss of biodiversity, droughts, floods, forest fires, ocean acidification, melting of north and south pole glaciers (NSPG), and sea-level rise (Maximillian et al. 2019; Mora et al. 2018; Yang et al. 2022).

In response to rising global greenhouse gas concentrations and temperatures, on December 12, 2015, 197 member parties of the United Nations framework convention on climate change (UNFCCC) unanimously agreed at the Paris climate change conference (PCCC) to adopt the Paris agreement, which lays out plans for global action to address climate change after 2020 (Berndes et al. 2016). Under the Paris agreement, each country agreed to limit the global temperature increase to less than 2 °C and work to limit the global temperature increase to less than 1.5 °C (Agreement 2015). As of February 2021, 124 countries worldwide have declared their intention to become carbon neutral and achieve net-zero carbon emissions by 2050 or 2060 (Chen 2021). To attain the targets stipulated by the Paris agreement and support sustainable development, it is necessary to not only reduce CO_2_ emissions but also to remove CO_2_ from the atmosphere to achieve net-zero carbon or negative carbon emissions through various social, economic, environmental and technological measures.

Carbon neutrality, a state of net-zero carbon emissions, can be achieved by balancing the total amount of carbon dioxide or greenhouse gas emissions produced directly or indirectly by a country, company, product, activity, or individual over a certain period via carbon offset or removal initiatives. Furthermore, to achieve carbon neutrality, the intergovernmental panel on climate change (IPCC), in its special report on global warming of 1.5 °C, also emphasized the need to reduce and phase out fossil fuels, use more renewable energy, improve energy efficiency, and highlighted the importance of implementing these measures in cities to achieve carbon neutrality (Masson-Delmotte et al. 2018). Moreover, to achieve net-zero carbon emissions and sustainable development, carbon removal or sequestration in terrestrial and marine ecosystems must be promoted (Cheng 2020). Different regions, countries, and cities have developed strategies to improve carbon removal or sequestration and achieve carbon neutrality (Hepburn et al. 2021; Pedersen et al. 2020; Huang and Zhai 2021), yet achieving net-zero carbon emissions is challenging (Wang et al. 2021).

Herein, this literature review presents a systematic discussion of the implications of the 26th summit of the United Nations Climate Change Conference of the Parties for achieving carbon neutrality, specifically addressing the implications of achieving such a target by 2050 or 2060 for most member countries. Furthermore, the review explores global initiatives, primarily referring to the policies or measures put in place by individual countries to achieve net-zero carbon emissions. The review also investigates in detail the interrelationships and synergies between adaptation and mitigation strategies, maps direct and indirect carbon emissions and proposes two main approaches to achieving carbon neutrality: emissions reduction and atmospheric carbon removal. Moreover, the review presents carbon-free plans for the future in transportation, agriculture, food waste, industry, and other areas and examines the life cycle analysis of various carbon neutral systems for the technologies or measures to achieve these plans. Finally, the review provides relevant and up-to-date information, policies, and technologies for achieving carbon neutrality and assists governments and people in different regions and countries in understanding the positive environmental, social, and economic consequences of carbon neutrality.

## United Nations Climate Change Conference of the Parties

### Background of the Conference

At a critical time for green recovery on a global scale, the 26th United Nations Climate Change Conference of the Parties was held in Glasgow from 31st October to 12th November 2021 (Masuda et al. 2022). It is well known that greenhouse gas emission reductions are a key factor in human health. The 2030 enhanced emission reduction targets (in the form of nationally determined contributions) and the mid-century long-term low greenhouse gas emission development strategies that the worldwide governments are supposed to submit to the 26th United Nations Climate Change Conference of the Parties have only been submitted by countries accounting for 55% and 32% of global greenhouse gas emissions, respectively (Wyns and Beagley 2021). At the 26th United Nations Climate Change Conference of the Parties, climate change moved from a marginal issue to a worldwide priority. With the attention of world leaders, government representatives, businesses and citizens focused on the 26th United Nations Climate Change Conference of the Parties in Glasgow, expectations are high for countries to make new commitments on reducing carbon emissions. In the meantime, it is essential to look back at another United Nations Climate Change Conference of the Parties. For the first time ever, a major event occurred in 2015. At the 21st United Nations Climate Change Conference of the Parties, every country agreed to combat the negative impacts of climate change by working together to keep global warming in a range well below 2 °C, with a target value of 1.5 °C (Vogler 2020). At the same time, each nation party agreed to provide funding to achieve these goals (van den Berg et al. 2022). This marked the birth of the Paris agreement.

### Outcomes of the Conference

**Fig. 1:**
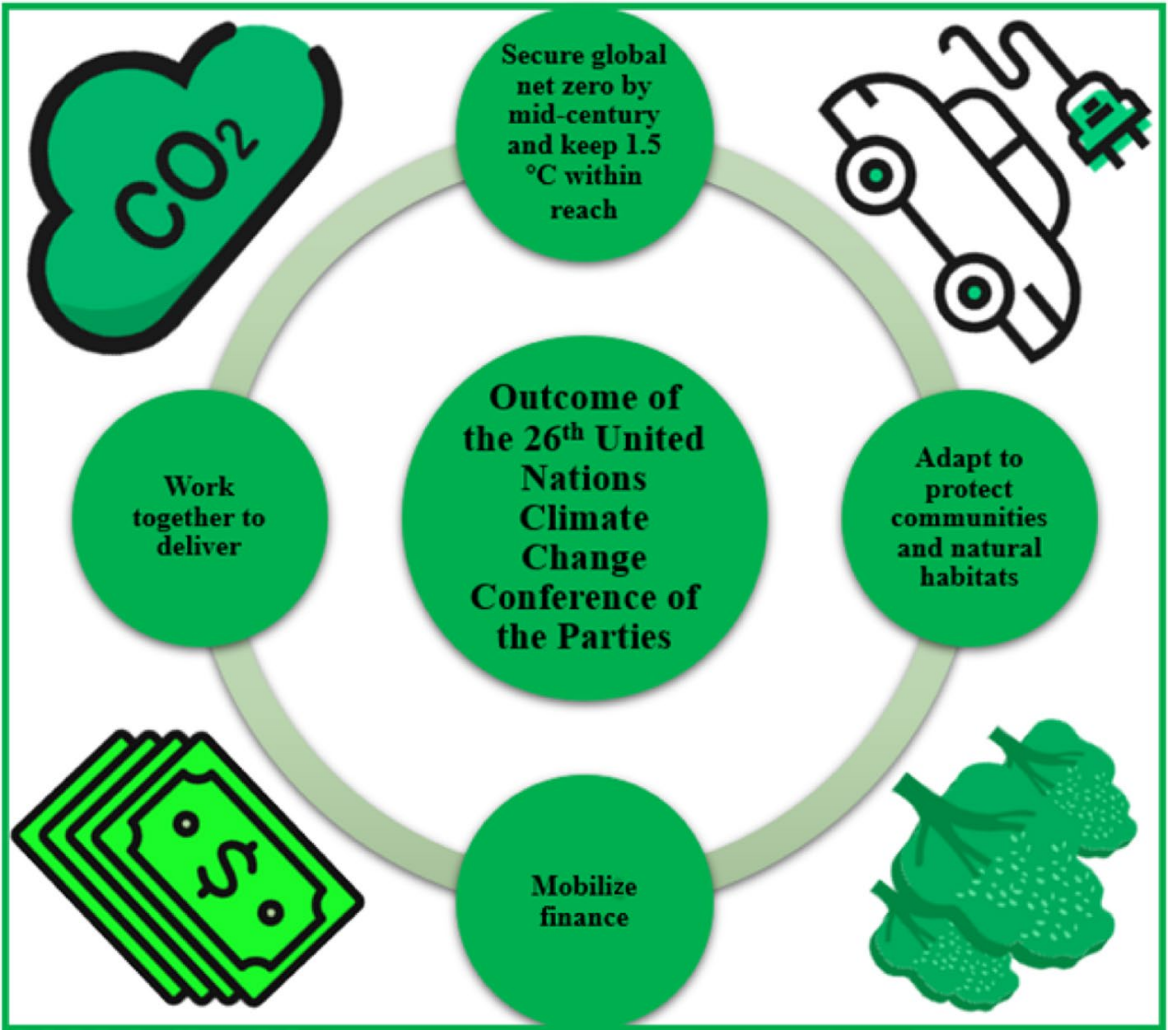
The main outcomes of the United Nations Climate Change Conference of the Parties. Figure 1 illustrates the four main outcome goals of the 26th United Nations Climate Change Conference of the Parties: secure global net-zero by mid-century and keep 1.5 °C within reach, adapt to protect communities and natural habitats, mobilize finance, and work together to deliver. These four outcome goals focus on coal, electric vehicles, cash, and trees

The 26th United Nations Climate Change Conference of the Parties made progress in four key areas: coal, cars, cash, and trees. Progress in the first two goals requires a consensus among countries to rapidly phase out coal, the most polluting fossil fuel. The second is to replace fuel-based transport with electric transport as soon as possible and to develop electric vehicles. Regarding the latter two goals, the $100 billion in annual financial support pledged by developed countries to developing countries in 2010 will have to be delivered. At the same time, climate change solutions, which are part of the biology of global change, should be implemented and delivered (Smith et al. 2022). Figure 1 presents the four main outcomes of the 26th United Nations Climate Change Conference of the Parties.

- Secure global net-zero by mid-century and keep 1.5 °C within reach

To avoid the looming problem of global environmental change, global warming needs to be limited to less than 1.5 °C. At present, the world has not yet limited global warming to 1.5 °C (Arasaradnam and Hillman 2022; COP26 2021; Dwivedi et al. 2022). Without targeted improvements, global temperatures will continue to rise, leading to more catastrophic floods, bushfires, extreme weather, and species destruction. Experts have made some progress in combating global warming, bending the temperature curve to 2 °C. Nevertheless, scientific data show that much work remains to be done to keep the temperature curve at 1.5 °C (Kelly 2021). Developed countries and those with large carbon emissions need to take the lead, and goals must be quickly translated into action. Countries worldwide (especially developed countries) must rapidly phase out fossil fuel power generation and provide support to developing countries for clean energy technologies (COP26 2021; Laybourn-Langton and Smith 2022). At the same time, cleaning up the air and reducing carbon emissions by shifting to zero-emission cars, vans and trucks are also very important factors (COP26 2021).

- Adapt to protect communities and natural habitats

People worldwide are already living in devastating climate scenarios as a result of global warming. Human security is at risk, and humankind must act and be proactive in addressing the severe challenges caused by climate change. Governments must unite to assist those most vulnerable. It is vital to take adequate precautionary measures to avoid or mitigate the damage caused by climate change. Simultaneously, building financial plans for early warning systems and robust infrastructure is critical. Protecting and restoring habitats is critical for mitigating the harmful consequences of climate change and addressing natural storm and flood management challenges (WHO 2021).

- Mobilize finance

In order to achieve the stated climate goals, every practitioner in the financial industry requires change. To reduce the negative impacts of climate change on residential life, governments need to provide a certain amount of funding to do this. Governments should provide greener, more climate-resilient infrastructure development and support technological innovation (Jacobs 2021). Developed countries need to provide assistance to developing countries and help translate much of the public funds into climate-resilient investments (dos Santos 2022). It is critical to note that businesses must understand the risks climate change poses to their operations and plan accordingly. National banks and regulators must ensure that local financial systems are resilient to climate change’s adverse effects and assist companies in transitioning to zero emissions.

- Work together to deliver

The agreement reached in the 26th United Nations Climate Change Conference of the Parties negotiations is a shared responsibility of the member parties towards a net-zero economy through national efforts. The 26th United Nations Climate Change Conference of the Parties negotiations are focused on the rules needed for the eventual implementation of the Paris agreement, known as the Paris rulebook. This would require cooperation at the global level among governments, national functional sectors, and financial institutions (Arora and Mishra 2021). Each country is expected to develop policies appropriate to its own circumstances and not just make commitments to the global citizens but to work together to face and solve the global problem of climate change (Buchanan et al. 2022). Governments must reach an agreement that drives the world to maintain a temperature of 1.5 °C in the coming years.

This section explained the four important goal outcomes of the 26th United Nations Climate Change Conference of the Parties. The outcomes of these four goals are very significant in guiding the worldwide efforts to address carbon emissions and enhance the participation of countries in achieving net-zero carbon emissions.

## Worldwide initiatives to achieve carbon neutrality

Environmental degradation and global warming are the most important ecological and environmental problems facing humanity in today’s world. Without effective initiatives, policies, and other measures taken by various countries worldwide, the deteriorating ecological environment will continue to affect future generations (Li et al. 2021). Due to the continuous use of fossil fuels, global carbon dioxide emissions have reached an unprecedented peak in 2020 (IEA 2021), which has significantly contributed to global warming. As a result, the increased awareness of reducing fossil fuel use has also contributed to the enactment of global climate agreements. One of them is the Paris climate agreement (Nations 2015), issued by the United Nations, which aims to keep global warming below 1.5 °C and states that each country needs to enact policies or develop measures to reduce carbon emissions effectively. Although the ultimate goal of this initiative is to achieve carbon neutrality in all countries, different regions, cities, and institutions have different initiatives, approaches, or measures to reduce carbon emissions.

In China, the Chinese government formulated the “guidance on accelerating the establishment of a sound green low-carbon circular development economic system”, which specifies reaching peak carbon by 2030, achieving carbon neutrality by 2060, and striving to gradually achieve net-zero CO_2_ emissions (SCPRC 2021; Zhao et al. 2022). In addition to this, in their study, Cheng et al. pointed out that some Nordic countries have developed and implemented Pigouvian tax mechanisms to help achieve carbon neutrality through tax policies (Cheng et al. 2021). A study by Sen et al. from Victoria University, Australia, suggested that the creation, expansion, and dissemination of knowledge and learning about carbon neutrality would help to achieve the country’s goal of achieving carbon neutrality eventually and that this initiative and policy is clearly reflected in university educational institutions (Sen et al. 2022). The development of initiatives, policies, and measures related to reducing greenhouse gas emissions in each country is essential to fully achieve the goal of carbon neutrality by 2050 or 2060. Therefore, Table 1 summarizes the relevant documents of various countries worldwide that have developed initiatives, laws, or referred to carbon neutrality in their policies for achieving carbon neutrality goals.

**Table 1:** Worldwide initiatives to achieve carbon neutrality by countries. In Table 1, N/A indicates not available, URL indicates uniform resource locator, and COP26 is the 26th United Nations Climate Change Conference of the Parties, which provides statistics on the status of different countries in achieving carbon neutrality and the specific year in the future in which this will be achieved

Initiative names	Country	End target year	Target Status	Status date	Source URL
Benin’s first nationally determined contribution under the Paris agreement	Benin	2000	Achieved (self-declared)	2020	https://cop25.mma.gob.cl/wp-content/uploads/2020/02/Annex-Alliance-ENGLISH.pdf
Kingdom of Bhutan intended nationally determined contribution	Bhutan	2000	Achieved (self-declared)	2020	https://www4.unfccc.int/sites/submissions/INDC/Published%20Documents/Bhutan/1/Bhutan-INDC-20150930.pdf
Enhanced ambition in national climate plans	Gabon	2000	Achieved (self-declared)	2020	https://cop25.mma.gob.cl/wp-content/uploads/2020/12/1312-Annex-Alliance-ENGLISH-VF-2012.pdf
Updated nationally determined contribution in the framework of the Paris climate agreement	Guinea-Bissau	2030	Achieved (self-declared)	2021	https://www4.unfccc.int/sites/ndcstaging/PublishedDocuments/Guinea-Bissau%20First/NDC-Guinea%20Bissau-12102021.Final.pdf
Nationally determined contribution	Guyana	2019	Achieved (self-declared)	2020	http://spappssecext.worldbank.org/sites/indc/PDF_Library/gy.pdf
Enhanced ambition in national climate plans	Cambodia	2000	Achieved (self-declared)	2020	https://cop25.mma.gob.cl/wp-content/uploads/2020/02/Annex-Alliance-ENGLISH.pdf
Intended nationally determined contributions	Liberia	2000	Achieved (self-declared)	2020	https://www4.unfccc.int/sites/ndcstaging/PublishedDocuments/Liberia%20First/INDC%20Final%20Submission%20Sept%2030%202015%20Liberia.pdf
Madagascar’s intended nationally determined contribution	Madagascar	2010	Achieved (self-declared)	2019	https://www4.unfccc.int/sites/ndcstaging/PublishedDocuments/Madagascar%20First/Madagascar%20INDC%20Eng.pdf
Nationally determined contribution 2020	Suriname	N/A	Achieved (self-declared)	2014	https://www4.unfccc.int/sites/ndcstaging/PublishedDocuments/Suriname%20Second/Suriname%20Second%20NDC.pdf
N/A	Congo	2030	Declaration/pledge	2020	https://ndcpartnership.org/countries-map/country?iso=COG
Climate action in Estonia: latest state of play	Estonia	2050	Declaration/pledge	2021	https://www.europarl.europa.eu/thinktank/de/document.html?reference=EPRS_BRI%282021%29690684
South Africa Low Emission Development Strategy 2050	South Africa	2050	Declaration/pledge	2020	https://unfccc.int/sites/default/files/resource/South%20Africa%27s%20Low%20Emission%20Development%20Strategy.pdf
Zimbabwe Revised Nationally Determined Contribution	Zimbabwe	2030	Declaration/pledge	2020	https://www4.unfccc.int/sites/ndcstaging/PublishedDocuments/Zimbabwe%20First/Zimbabwe%20Revised%20Nationally%20Determined%20Contribution%202021%20Final.pdf
Andorran Nationally Determined Contribution	Andorra	2050	Declaration/pledge	N/A	https://www4.unfccc.int/sites/ndcstaging/PublishedDocuments/Andorra%20First/20200514-%20Actualitzaci%C3%B3%20NDC.pdf
Second Nationally Determined Contribution of the United Arab Emirates	United Arab Emirates	2050	Declaration/pledge	2021	https://www4.unfccc.int/sites/ndcstaging/PublishedDocuments/United%20Arab%20Emirates%20Second/UAE%20Second%20NDC%20-%20UNFCCC%20Submission%20-%20English%20-%20FINAL.pdf
Australia’s nationally determined contribution communication 2021	Australia	2050	Declaration/pledge	2021	https://www4.unfccc.int/sites/ndcstaging/PublishedDocuments/Australia%20First/Australia%20Nationally%20Determined%20Contribution%20Update%20October%202021%20WEB.pdf
Bahrain pledges to reach net zero emissions by 2060	Bahrain	2060	Declaration/pledge	2021	https://www.arabianbusiness.com/industries-energy/470085-bahrain-pledges-to-reach-net-zero-emissions-by-2060
Nationally determined contribution key parameters	Côte d’Ivoire	2030	Declaration/pledge	N/A	https://www.ndcs.undp.org/content/ndc-support-programme/en/home/our-work/geographic/africa/CotedIvoire.html#:~:text=NDC%20KEY%20PARAMETERS,management%20and%20recovery%20of%20waste
Contribution determinee au niveau national—actualisee	Cameroon	2030	Declaration/pledge	2021	https://www4.unfccc.int/sites/ndcstaging/PublishedDocuments/Cameroon%20First/CDN%20r%C3%A9vis%C3%A9e%20CMR%20finale%20sept%202021.pdf
N/A	Ghana	N/A	Declaration/pledge	N/A	https://www4.unfccc.int/sites/NDCStaging/Pages/Party.aspx?party=GHA
PM Modi sets India’s 2070 zero carbon emission target at COP26 summit	India	2070	Declaration/pledge	2021	https://www.hindustantimes.com/world-news/pm-modi-sets-india-2070-zero-carbon-emission-target-at-cop26-summit-101635785945035.html
COP26: Israel to hit zero net emissions by 2050, Bennett pledges	Israel	2050	Declaration/pledge	2021	https://www.jpost.com/climate-change/cop26-israel-to-aim-for-zero-net-emissions-by-2050-683470
N/A	Kazakhstan	2050	Declaration/pledge	2020	https://www.climateambitionsummit2020.org/ondemand.php
Twelfth Malaysia Plan	Malaysia	2050	Declaration/pledge	2021	https://rmke12.epu.gov.my/bm
Nigeria Pledges to Reach Net-Zero Emissions by 2060, Buhari Says	Nigeria	2060	Declaration/pledge	2021	https://www.bloomberg.com/news/articles/2021-11-02/nigeria-targets-to-reach-net-zero-emissions-by-2060-buhari-says
The government is instructed to limit greenhouse gas emissions and approve the country’s low-carbon development strategy	Russian Federation	2060	Declaration/pledge	2021	https://www.economy.gov.ru/material/news/pravitelstvu_porucheno_ogranichit_vybrosy_parnikovyh_gazov_i_utverdit_strategiyu_nizkouglerodnogo_razvitiya_strany.html
Saudi Arabia Commits to Net-Zero Emissions by 2060	Saudi Arabia	2060	Declaration/pledge	2021	https://www.bloomberg.com/news/articles/2021-10-23/world-s-biggest-oil-exporter-commits-to-net-zero-emissions
An Ambitious, Stakeholder-Driven Climate Change Commitment Ahead of COP26: Eswatini’s Revised Nationally Determined Contribution Process	Eswatini	N/A	Declaration/pledge	N/A	http://www.ipsnews.net/2021/10/ambitious-stakeholder-driven-climate-change-commitment-ahead-cop26-eswatinis-revised-nationally-determined-contribution-ndc-process/
N/A	Thailand	2050	Declaration/pledge	2021	https://www.youtube.com/watch?v=xiu_91tJa0o
Viet Nam to take stronger measures to achieve net-zero emissions by 2050	Vietnam	2050	Declaration/pledge	2021	http://news.chinhphu.vn/Home/Viet-Nam-to-take-stronger-measures-to-achieve-netzero-emissions-by-2050/202111/46000.vgp
Net-Zero Emissions by 2050	Canada	2050	In law	2021	https://www.canada.ca/en/services/environment/weather/climatechange/climate-plan/net-zero-emissions-2050.html
Net-Zero Emissions by 2050	Germany	2045	In law	2021	https://www.bundesregierung.de/breg-de/themen/klimaschutz/climate-change-act-2021-1936846
During the Conference of the Parties, Denmark passes Climate Act with a 70 percent reduction target	Denmark	2050	In law	2020	https://en.kefm.dk/news/news-archive/2019/dec/during-the-cop-denmark-passes-climate-act-with-a-70-percent-reduction-targetws-page-eng
Consolidated legislation on climate change and energy transition	Spain	2050	In law	2021	https://boe.es/buscar/act.php?id=BOE-A-2021-8447#top
Law on Energy and Climate	France	2050	In law	2020	https://www.legifrance.gouv.fr/affichTexte.do?cidTexte=JORFTEXT000039355955&categorieLien=id
Net Zero Strategy: build Back Greener	United Kingdom	2050	In law	2020	https://www.gov.uk/government/publications/net-zero-strategy
On the debate on the commission amendment to the bill on the declaration of the climate emergency	Hungary	2050	In law	2020	https://www.parlament.hu/irom41/07021/07021-0010.pdf
Climate action 2019 to tackle climate breakdown	Ireland	2050	In law	2021	https://www.gov.ie/pdf/?file=https://assets.gov.ie/42213/752d5346b9c6407b9125fdadfa0738a4.pdf#page=1
Japan’s Greenhouse Gas Emission Reduction Target	Japan	2050	In law	2021	https://www4.unfccc.int/sites/ndcstaging/PublishedDocuments/Japan%20First/JAPAN_FIRST%20NDC%20(INTERIM-UPDATED%20SUBMISSION).pdf
The Republic of Korea’s Update of its First Nationally Determined Contribution	South Korea	2050	In law	2021	https://www4.unfccc.int/sites/ndcstaging/PublishedDocuments/Republic%20of%20Korea%20First/201230_ROK’s%20Update%20of%20its%20First%20NDC_editorial%20change.pdf
N/A	Norway	2050	In law	2020	https://www4.unfccc.int/sites/NDCStaging/pages/Party.aspx?party=NOR
Climate change response (zero-carbon) amendment act 2019	New Zealand	2050	In law	2020	https://www.legislation.govt.nz/act/public/2019/0061/latest/LMS183848.html
long-term low greenhouse gas emission development strategy of the European Union and its member states	Portugal	2045	In law	2021	https://unfccc.int/sites/default/files/resource/HR-03-06-2020%20EU%20Submission%20on%20Long%20term%20strategy.pdf
The Swedish climate policy framework	Sweden	2045	In law	2018	https://www.government.se/495f60/contentassets/883ae8e123bc4e42aa8d59296ebe0478/the-swedish-climate-policy-framework.pdf
Expected and nationally determined contribution	Guatemala	2030	In law	2020	https://www4.unfccc.int/sites/ndcstaging/PublishedDocuments/Guatemala%20First/Gobierno%20de%20Guatemala%20INDC-UNFCCC%20Sept%202015.pdf
Climate change	Netherlands	2050	In law	2019	https://www.rijksoverheid.nl/onderwerpen/klimaatverandering/klimaatbeleid
2050 long-term strategy	European Union	2050	In law	2020	https://ec.europa.eu/clima/eu-action/climate-strategies-targets/2050-long-term-strategy_en
Antigua and Barbuda updated nationally determined contribution	Antigua and Barbuda	2040	In policy document	2020	https://www4.unfccc.int/sites/ndcstaging/PublishedDocuments/Antigua%20and%20Barbuda%20First/ATG%20-%20UNFCCC%20NDC%20-%202021-09-02%20-%20Final.pdf
Integrated national energy and climate plan for Austria	Austria	2040	In policy document	2020	https://ec.europa.eu/energy/sites/ener/files/documents/at_final_necp_main_en.pdf
Belize updated nationally determined contribution	Belize	2050	In policy document	2021	https://www4.unfccc.int/sites/ndcstaging/PublishedDocuments/Belize%20First/Belize%20Updated%20NDC.pdf
Barbados’ second national communication under the United Nations framework convention on climate change	Barbados	2030	In policy document	2020	https://www4.unfccc.int/sites/SubmissionsStaging/NationalReports/Documents/4693851_Barbados-NC2-1-Barbados%20SNC%20FINAL%20April%202018.pdf
Chile’s nationally determined contribution	Chile	2050	In policy document	2020	https://www4.unfccc.int/sites/ndcstaging/PublishedDocuments/Chile%20First/Chile%27s_NDC_2020_english.pdf
China’s mid-century long-term low greenhouse gas emission development strategy	China	2060	In policy document	2020	https://unfccc.int/sites/default/files/resource/China%E2%80%99s%20Mid-Century%20Long-Term%20Low%20Greenhouse%20Gas%20Emission%20Development%20Strategy.pdf
Nationally determined contribution key parameters	Dem. Rep. Congo	2030	In policy document	2015	https://www.ndcs.undp.org/content/ndc-support-programme/en/home/our-work/geographic/africa/DRC.html
National decarbonization plan	Costa Rica	2050	In policy document	2020	https://cambioclimatico.go.cr/wp-content/uploads/2020/01/NationalDecarbonizationPlan.pdf
Climate action in Czechia	Czech Republic	2030	In policy document	2020	https://www.europarl.europa.eu/RegData/etudes/BRIE/2021/689329/EPRS_BRI(2021)689329_EN.pdf
Intended nationally determined contribution of the Republic of Djibouti	Djibouti	2030	In policy document	2016	https://www.climatewatchdata.org/ndcs/country/DJI/full?document=first_ndc
Intended nationally determined contribution of the Commonwealth of Dominica	Dominica	2030	In policy document	2016	https://www4.unfccc.int/sites/ndcstaging/PublishedDocuments/Dominica%20First/Commonwealth%20of%20Dominica-%20Intended%20Nationally%20Determined%20Contributions%20(INDC).pdf
Ministry launches the Ecuador zero carbon program	Ecuador	2050	In policy document	2020	https://www.ambiente.gob.ec/ministerio-pone-en-marcha-el-programa-ecuador-carbono-cero/
Finland's national climate change policy	Finland	2035	In policy document	2015	https://ym.fi/en/finland-s-national-climate-change-policy
Fiji low emission development strategy 2018–2050	Fiji	2050	In policy document	2020	https://unfccc.int/sites/default/files/resource/Fiji_Low%20Emission%20Development%20%20Strategy%202018%20-%202050.pdf
Climate change mitigation and adaptation	Greece	2050	In policy document	2020	https://www.oecd-ilibrary.org/sites/ff34a34b-en/index.html?itemId=/content/component/ff34a34b-en
Low-carbon development strategy of the Republic of Croatia until 2030 with a view to 2050	Croatia	2050	In policy document	2020	https://mingor.gov.hr/UserDocsImages/klimatske_aktivnosti/odrzivi_razvoj/NUS/lts_nus_eng.pdf
Iceland’s 2020 climate action plan	Iceland	2040	In policy document	2020	https://www.government.is/library/01-Ministries/Ministry-for-The-Environment/201004%20Umhverfisraduneytid%20Adgerdaaaetlun%20EN%20V2.pdf
Long-term Italian strategy on reducing greenhouse gas emissions	Italy	2050	In policy document	2021	https://ec.europa.eu/clima/sites/lts/lts_it_it.pdf
Updated nationally determined contribution	Saint Kitts and Nevis	2030	In policy document	2021	https://www4.unfccc.int/sites/ndcstaging/PublishedDocuments/Saint%20Kitts%20and%20Nevis%20First/St.%20Kitts%20and%20Nevis%20Revised%20NDC_Updated.pdf
Saint Lucia’s updated nationally determined contribution communicated to the United Nations framework convention on climate change	Saint Lucia	2030	In policy document	2016	https://www4.unfccc.int/sites/ndcstaging/PublishedDocuments/Saint%20Lucia%20First/Saint%20Lucia%20First%20NDC%20(Updated%20submission).pdf
Environmental performance reviews: Lithuania 2021	Lithuania	2050	In policy document	2020	https://www.oecd-ilibrary.org/environment/oecd-environmental-performance-reviews-lithuania-2021_48d82b17-en
Luxembourg’s integrated national energy and climate plan for 2021–2030	Luxembourg	2050	In policy document	2019	https://ec.europa.eu/energy/sites/ener/files/documents/lu_final_necp_main_en.pdf
Intended nationally determined contribution of the European Union and its member states	Latvia	2050	In policy document	2020	https://www4.unfccc.int/sites/ndcstaging/PublishedDocuments/Austria%20First/LV-03-06-EU%20INDC.pdf
15 world leaders commit to delivering new Paris targets by early 2020 and to achieving net-zero global emissions by 2050 on eve of the United Nations summit	Monaco	2050	In policy document	2020	https://www.docdroid.net/gavlB6o/190922-rmi-unsg-summit-release-leaders-statement-final-combined-pdf
Update of nationally determined contribution of Maldives	Maldives	2030	In policy document	2020	https://www4.unfccc.int/sites/ndcstaging/PublishedDocuments/Maldives%20First/Maldives%20Nationally%20Determined%20Contribution%202020.pdf
Tile Til Eo 2050 climate strategy “lighting the way”	Marshall Islands	2050	In policy document	2020	https://unfccc.int/sites/default/files/resource/180924%20rmi%202050%20climate%20strategy%20final_0.pdf
Malta low carbon development strategy	Malta	2050	In policy document	2020	https://meae.gov.mt/en/Public_Consultations/MECP/PublishingImages/Pages/Consultations/MaltasLowCarbonDevelopmentStrategy/Malta%20Low%20Carbon%20Development%20Strategy.pdf
On Slovenia’s long-term climate strategy until 2050	Slovenia	2050	In policy document	2020	https://unfccc.int/sites/default/files/resource/LTS1_SLOVENIA_EN.pdf
Enhanced ambition in national climate plans	Uruguay	2050	In policy document	2020	https://cop25.mma.gob.cl/wp-content/uploads/2020/02/Annex-Alliance-ENGLISH.pdf
Pathways to net-zero greenhouse gas emissions by 2050	United States of America	2050	In policy document	2021	https://www.whitehouse.gov/wp-content/uploads/2021/10/US-Long-Term-Strategy.pdf
Intended nationally determined contribution of the Republic of Albania following decision	Albania	2030	In policy document	N/A	https://www4.unfccc.int/sites/ndcstaging/PublishedDocuments/Albania%20First/Albania%20First.pdf
N/A	Azerbaijan	2030	In policy document	2017	https://zerotracker.net/
N/A	Belarus	2030	In policy document	N/A	https://eu4climate.eu/belarus/
Government of Bermuda—protecting the environment	Bermuda	2035	In policy document	N/A	https://www.gov.bm/articles/government-bermuda-%E2%80%93-protecting-environment
Paris agreement Brazil’s nationally determined contribution	Brazil	2060	In policy document	2020	https://www4.unfccc.int/sites/ndcstaging/PublishedDocuments/Brazil%20First/Brazil%20First%20NDC%20(Updated%20submission).pdf
Summary of the first nationally determined contribution updated (2020–2030)	Cuba	2030	In policy document	2020	https://www4.unfccc.int/sites/ndcstaging/PublishedDocuments/Cuba%20First/Cuban%20First%20NDC%20Summary%20(Updated%20submission).pdf
N/A	Algeria	2030	In policy document	N/A	https://zerotracker.net/
Egyptian intended nationally determined contribution	Egypt	2030	In policy document	2017	https://www4.unfccc.int/sites/ndcstaging/PublishedDocuments/Egypt%20First/Egyptian%20INDC.pdf
Irap nationally determined contribution	Iraq	2030	In policy document	2021	https://www4.unfccc.int/sites/ndcstaging/PublishedDocuments/Iraq%20First/Iraq%20NDC%20Document.docx
Updated submission of Jordan’s 1^st^ nationally determined contribution	Jordan	2030	In policy document	2021	https://www4.unfccc.int/sites/ndcstaging/PublishedDocuments/Jordan%20First/UPDATED%20SUBMISSION%20OF%20JORDANS.pdf
Submission of Kenya’s updated nationally determined contribution	Kenya	2030	In policy document	2020	https://www4.unfccc.int/sites/ndcstaging/PublishedDocuments/Kenya%20First/Kenya’s%20First%20%20NDC%20(updated%20version).pdf
The Kyrgyz Republic intended nationally determined contribution	Kyrgyzstan	2050	In policy document	2015	https://www4.unfccc.int/sites/ndcstaging/PublishedDocuments/Kyrgyzstan%20First/Kyrgyzstan%20INDC%20_ENG_%20final.pdf
Sri Lanka updated nationally determined contribution	Sri Lanka	2060	In policy document	2021	https://www4.unfccc.int/sites/ndcstaging/PublishedDocuments/Sri%20Lanka%20First/NDCs%20of%20Sri%20Lanka-2021.pdf
Nationally determined contribution—updated	Morocco	2030	In policy document	2021	https://www4.unfccc.int/sites/ndcstaging/PublishedDocuments/Morocco%20First/Moroccan%20updated%20NDC%202021%20_Fr.pdf
Republic of Moldova’s intended national determined contribution	Moldova, Republic of	2030	In policy document	2020	https://www4.unfccc.int/sites/ndcstaging/PublishedDocuments/Republic%20of%20Moldova%20First/INDC_Republic_of_Moldova_25.09.2015.pdf
Enhanced nationally determined contribution	Macedonia, the former Yugoslav Republic of	2030	In policy document	2021	https://www4.unfccc.int/sites/ndcstaging/PublishedDocuments/The%20Republic%20of%20North%20Macedonia%20First/Macedonian%20enhanced%20NDC%20(002).pdf
Updated nationally determined contribution	Panama	2050	In policy document	2021	https://www4.unfccc.int/sites/ndcstaging/PublishedDocuments/Panama%20First/CDN1%20Actualizada%20Rep%C3%BAblica%20de%20Panam%C3%A1.pdf
Nationally determined contribution communicated to the United Nations framework convention on climate change	Philippines	2030	In policy document	2021	https://www4.unfccc.int/sites/ndcstaging/PublishedDocuments/Philippines%20First/Philippines%20-%20NDC.pdf
Updated nationally determined contribution of the Democratic People’s Republic of Korea	North Korea	2030	In policy document	2019	https://www4.unfccc.int/sites/ndcstaging/PublishedDocuments/Democratic%20People’s%20Republic%20of%20Korea%20First/2019.09.19_DPRK%20letter%20to%20SG%20special%20envoy%20for%20NDC.pdf
Update of the nationally determined contribution of the Republic of Paraguay	Paraguay	2030	In policy document	N/A	http://www.mades.gov.py/wp-content/uploads/2021/07/ACTUALIZACION-DE-LA-NDC-DEL-PARAGUAY_Borrador-final_Julio-2021-1.pdf
The State of Palestine’s first nationally determined contributions “updated submission”	Palestinian Territory, Occupied	2040	In policy document	2021	https://www4.unfccc.int/sites/ndcstaging/PublishedDocuments/State%20of%20Palestine%20First/Updated%20NDC_%20State%20of%20Palestine_2021_FINAL.pdf
N/A	Qatar	2030	In policy document	2021	https://www4.unfccc.int/sites/ndcstaging/Pages/Party.aspx?party=QAT&prototype=1
San Marino’s intended nationally determined contribution	San Marino	2030	In policy document	2015	https://www4.unfccc.int/sites/ndcstaging/PublishedDocuments/San%20Marino%20First/SAN%20MARINO%20INDC%20EN.pdf
Intended nationally determined contribution	Turkey	2053	In policy document	2021	https://www2.tbmm.gov.tr/d27/2/2-3853.pdf
Updated nationally determined contribution of Ukraine to the Paris agreement	Ukraine	2060	In policy document	N/A	https://www4.unfccc.int/sites/ndcstaging/PublishedDocuments/Ukraine%20First/Ukraine%20NDC_July%2031.pdf
Republic of Uzbekistan updated nationally determined contribution	Uzbekistan	2030	In policy document	2021	https://www4.unfccc.int/sites/ndcstaging/PublishedDocuments/Uzbekistan%20First/Uzbekistan_Updated%20NDC_2021_EN.pdf
First nationally determined contribution of the Bolivarian Republic of Venezuela for the fight against climate change and its effects	Venezuela, Bolivarian Republic of	2030	In policy document	2015	https://www4.unfccc.int/sites/ndcstaging/PublishedDocuments/Venezuela%20(Bolivarian%20Republic%20of)%20First/Primera%20%20NDC%20Venezuela.pdf
Singapore’s climate action	Singapore	N/A	In policy document	2020	https://www.nccs.gov.sg/docs/default-source/publications/nccsleds.pdf
N/A	Target proposed/In discussion/Not available	Afghanistan, Angola, Argentina, Armenia, Belgium, Burkina Faso, Bangladesh, The Bahamas, Central African Republic, Switzerland, Colombia, Comoros, Cape Verde, Cyprus, Dominican Republic, Eritrea, Ethiopia, Micronesia, Guinea, The Gambia, Grenada, Haiti, Jamaica, Kiribati, Laos, Lebanon, Lesotho, Mexico, Mali, Myanmar, Mozambique, Mauritania, Mauritius, Malawi, Namibia, Niger, Nicaragua, Nepal, Nauru, Pakistan, Peru, Palau, Papua New Guinea, Rwanda, Senegal, Solomon Islands, Sierra Leone, Sao Tome and Principe, Slovakia, Seychelles, Chad, Togo, Timor-Leste, Tonga, Trinidad and Tobago, Tuvalu, Uganda, Saint Vincent and the Grenadines, Vanuatu, Samoa, Yemen, Zambia, Burundi, Bulgaria, Bosnia and Herzegovina, Bolivia, Brunei Darussalam, Botswana, Cayman Islands, Georgia, Equatorial Guinea, Honduras, Indonesia, Iran, Islamic Republic of Kuwait, Libya, Liechtenstein, Montenegro, Mongolia, Oman, Poland, Romania, Sudan, El Salvador, Somalia, Serbia, Syrian Arab Republic, Tajikistan, Turkmenistan, Tunisia, Tanzania, South Sudan, Niue (Total of 93 countries) (TRACKER, 2022)			

According to Table 1, a global count of 198 countries on initiatives to achieve carbon neutrality, we find that as of February 2022, all of these countries are committed to achieving carbon neutrality in the future, with Benin, Bhutan, Gabon, Guinea-Bissau, Guyana, Cambodia, Liberia, Madagascar, and Suriname already have achieved carbon neutrality. In addition, 21 countries have declared or committed to be carbon neutral between 2030 and 2070; this includes Congo, Estonia, South Africa, Zimbabwe, Andorra, United Arab Emirates, Australia, Bahrain, Côte d’Ivoire, Cameroon, Ghana, India, Israel, Kazakhstan, Malaysia, Nigeria, Russian Federation, Saudi Arabia, Eswatini, Thailand, and Vietnam. Additionally, 17 countries have proposed carbon-neutral legislation, and these countries are Canada, Germany, Denmark, Spain, France, United Kingdom, Hungary, Ireland, Japan, South Korea, Norway, New Zealand, Portugal, Sweden, Guatemala, Netherlands, and European Union. Furthermore, 58 countries have mentioned carbon neutrality targets in their policy documents, including Antigua and Barbuda, Austria, Belize, Barbados, Chile, China, Dem. Rep. Congo, Costa Rica, Czech Republic, Djibouti, Dominica, Ecuador, Finland, Fiji, Greece, Croatia, Iceland, Italy, Saint Kitts and Nevis, Saint Lucia, Lithuania, Luxembourg, Latvia, Monaco, Maldives, Marshall Islands, Malta, Slovenia, Uruguay, United States of America, Albania, Azerbaijan, Belarus, Bermuda, Brazil, Cuba, Algeria, Egypt, Iraq, Jordan, Kenya, Kyrgyzstan, Sri Lanka, Morocco, Moldova, Republic of Macedonia, the former Yugoslav Republic of Panama, Philippines, North Korea, Paraguay, Palestinian Territory, Qatar, San Marino, Turkey, Ukraine, Uzbekistan, Venezuela, the Bolivarian Republic of, and Singapore. In addition, 93 countries, including Afghanistan, Angola, Argentina, Armenia, Belgium, Burkina Faso, Bangladesh, and The Bahamas, along with others, are proposing or discussing documents related to achieving carbon neutrality targets (see Table 1).

Overall, of the 198 countries that have committed to achieving carbon neutrality goals, 4.5% have already achieved carbon neutrality, 10.6% have declared or committed to achieving carbon neutrality goals, 8.6% have legislated for achieving carbon neutrality goals, 29.3% have formulated relevant policies to achieve carbon neutrality goals, and the remaining 47% are in the process of discussing relevant documents to achieve carbon neutrality. In addition, 120 out of 198 countries, or 60.6%, aim to achieve carbon neutrality by 2050–2070. Based on the analysis of 198 countries worldwide on carbon neutrality initiatives, we found that most countries are discussing the development of documents related to achieving carbon neutrality, and most countries aim to achieve carbon neutrality after 2050.

## Interrelationships and synergies between adaptation and mitigation strategies

**Fig. 2:**
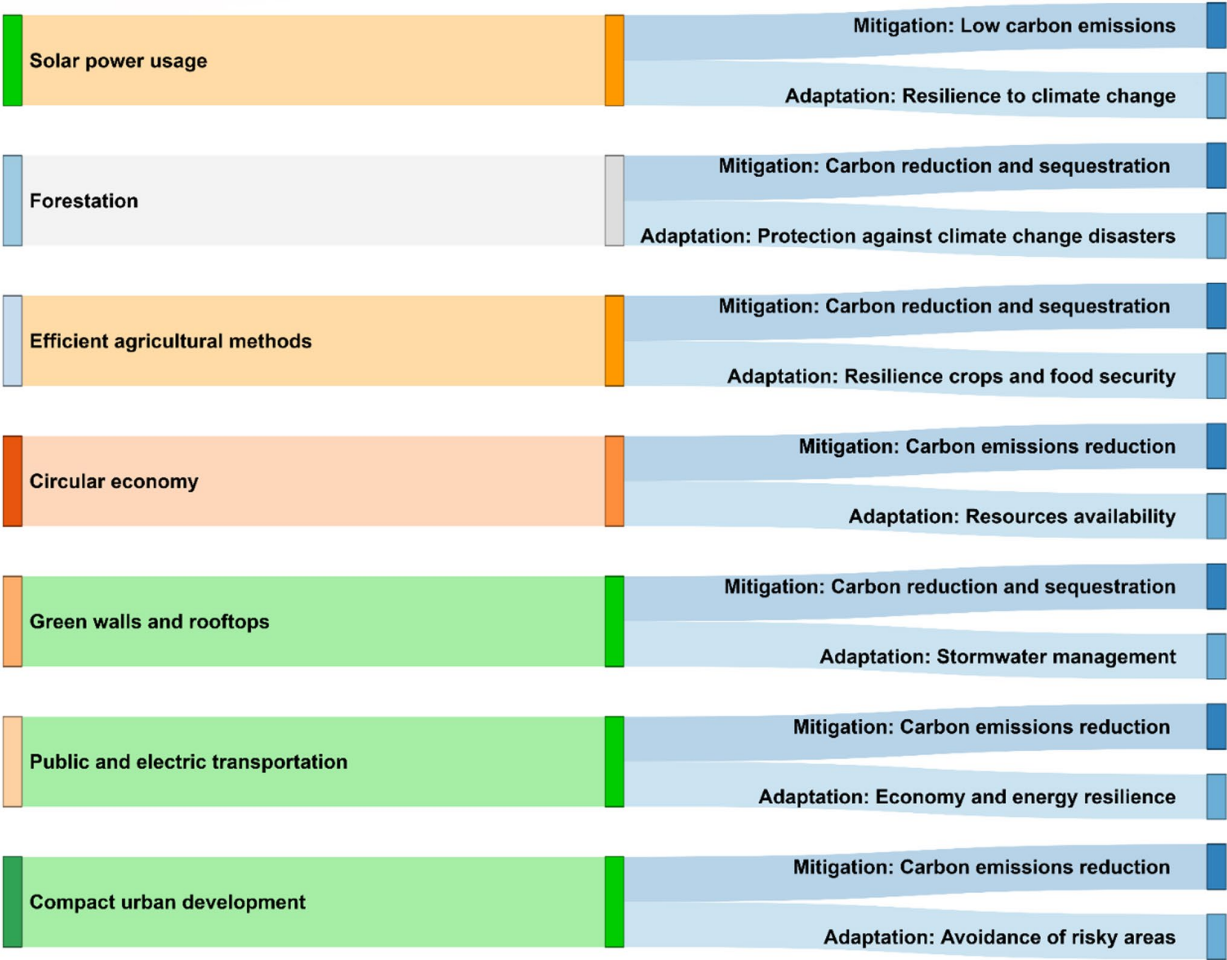
A summary of how interrelationships and synergies between mitigation and adaptation strategies co-benefit each other. For example, the usage of solar power for electricity or heating lowers carbon emissions as solar power is a renewable energy source hence mitigating climate change. Additionally, the usage of solar power adapts to climate change as solar power is resilient to climate change problems like storms and high temperatures, unlike the centralized grid systems that are vulnerable. The authors recommend that new carbon neutrality policies focus on mitigation and adaptation together rather than mitigation alone

While climate change mitigation strategies are critical, adaptation strategies are also essential. Historically, policymakers separated adaptation and mitigation strategies. However, there has been a recent trend toward investigating synergies between adaptation and mitigation techniques. The synergies are beneficial more than separate treatment of adaptation and mitigation (Fig. 2). A mitigation strategy of implementing distributed solar power in buildings instead of fossil fuel energy leads to low carbon emissions in the energy sector. The use of distributed solar power is in synergy with adaptation, as solar power leads to a more resilient power supply system than over-the-ground grids that are vulnerable to storms and temperature changes caused by climate change (Ripple et al. 2022). In nature, the planting and maintenance of forests is a synergy between mitigation and adaptation strategies. The forests mitigate climate change by reducing and storing carbon. In addition, the forests adapt to climate change by offering protection to droughts, fires, floods, and heatwaves (Moomaw et al. 2019). Other examples of energy and nature sector strategies in synergy and benefit both mitigation and adaptation are wind energy and urban green spaces.

Reduced forest conversion to agricultural land through the promotion of agroforestry, regenerative agriculture, and polyculture contributes to climate change mitigation and adaptation in the agricultural sector (Montanaro et al. 2018). Reduced forest conversion helps mitigate climate change by lowering greenhouse gas emissions and increasing carbon storage. Additionally, improving efficient agricultural practices aids in climate change adaptation by increasing soil carbon and water efficiency, resulting in resilient crops and food security. A transition from a linear to a circular economy, in which end-of-life goods can be used to create new goods, is one strategy to mitigate and adapt to climate change. Government initiatives such as carbon taxes, zero-carbon industry incentives, and mitigation and adaptation policies can all contribute to the circular economy’s transformation.

Constructing green walls and rooftops are one method of mitigating and adapting to climate change in buildings (Grafakos et al. 2019). Green walls and rooftops can mitigate climate change by reducing heat islands, lowering energy usage, and sequestering carbon. Additionally, green roofs increase stormwater management, allowing for adaptation to climate change-related flooding. Additional examples of agricultural, economic, and building sector methods that work in tandem and assist both mitigation and adaptation include genetically enhanced crops, funding net-zero carbon regulations, and geothermal energy use.

Promoting public transportation, increasing vehicle efficiency, electrifying transportation, and encouraging car-sharing services are all approaches to mitigate and adapt to climate change in the transportation sector (Sharifi 2021). All of these measures will reduce carbon emissions, ultimately mitigating climate change; simultaneously, they will result in cost and energy savings, thereby increasing economic and energy resilience and thus enabling climate change adaptation. In urban design, compact urban development with an appropriate density, land use mix, and accessibility contributes to climate change mitigation and adaptation. Compact urban development reduces per capita travel demand, energy demand for heating and cooling, and provides energy systems that are more efficient, so lowering carbon emissions and mitigating climate change. Additionally, compact urban development decreases land demand, avoids risky locations, and is less susceptible to intense heat events than urban sprawl, allowing for climate change adaptation. Congestion pricing and water-sensitive urban designs are two other examples of transportation and urban design sector strategies in synergy and enhance both mitigation and adaptation.

The synergies and trade-offs between mitigation and adaptation must be implemented carefully to not adversely influence one another. Positive synergies are mitigation measures that do not increase vulnerability and adaptation measures that do not increase greenhouse gas emissions (Zhao et al. 2018). For example, afforestation creates a beneficial synergy since afforestation works as a carbon sink and protects from calamities. Certain mitigation and adaptation techniques include trade-offs with unfavourable consequences. For instance, constructing a hydroelectric power plant will reduce greenhouse gas emissions due to its renewable energy source. However, a hydroelectric power plant will increase competition for water with local communities, compromising adaptation. On the other hand, while constructing a dam to prevent seawater intrusion will secure water supply and so reduce vulnerability, dam construction will generate greenhouse gases as a result of the cement and steel needed in construction, thereby impairing mitigation. Prior to implementing mitigation and adaptation techniques, policymakers should conduct an in-depth analysis to ensure that co-benefits are realized rather than negative impacts.

The integration of climate mitigation and adaptation in the European cities of Copenhagen and Helsinki was investigated (Landauer et al. 2018). The study concentrated on two contexts: (1) urban densification and buildings’ energy management for mitigation, and (2) urban heat and runoff management for adaptation. Synergies have been discovered in Copenhagen between energy efficiency and flood protection criteria for building design. Furthermore, the study discovered a contradiction in which a higher capacity of groundwater pumps was necessary to regulate floodwater, resulting in increased energy consumption. In Helsinki, strong national policies on energy-efficient building design compelled municipal governments to prioritize mitigation measures such as building insulation over adaptation measures such as using durable materials to safeguard buildings from flooding. The authors advise that a better knowledge of cross-scale interactions be developed to minimize conflicts and maximize the synergies of mitigation and adaptation efforts.

Grafakos et al. researched the integration of mitigation and adaptation in European cities by assessing 885 climate change action plans, of which only 147 had considered both mitigation and adaptation policies. The research showed that about 50% of climate change action plans address adaptation and mitigation by considering both greenhouse gas emissions and vulnerability profiles initial assessments (Grafakos et al. 2020). However, only a quarter of the climate change action plans consider an in-depth analysis of the mitigation and adaptation synergies and co-benefits. The sectors with the most synergies were green urban infrastructures, construction, energy efficiency, and buildings. Another study used a qualitative method to examine the policy implementation of Cameroon’s climate mitigation and adaptation initiatives (Ngum et al. 2019). While several policies address climate change, the findings indicated that they are all focused on mitigation rather than adaptation. Several constraints to synergies include a lack of finance, collaboration, implementation, transparency, and public engagement. Synergies can be achieved by forming a technical committee to advise the government on scientific issues related to climate change, private sector investment, community awareness, and collaboration with other countries that have experience with climate change mitigation and adaptation synergies.

Overall, the interrelationships and synergies between mitigation and adaptation methods, as well as their co-benefits, were discussed. Additionally, the detrimental impacts of certain strategies were demonstrated. The implication of synergies in different countries was shown not to progress well. For example, in Europe, only a quarter of the climate change action plans considered an in-depth analysis of the mitigation and adaptation synergies. In Cameroon, climate change initiatives were solely focused on mitigating the effects of climate change. Finally, methods for promoting mitigation and adaptation synergies were recommended, including investments and community awareness.

## Mapping direct and indirect carbon emissions

Carbon emissions mapping using statistical approaches is critical for determining the magnitude of emissions and developing strategies for reducing them in order to attain carbon neutrality (Table 2). Research was conducted on mapping CO_2_ emissions of various industrial sectors in China (Bai et al. 2018). The findings indicated that the majority of CO_2_ exporters are involved in (1) the production and supply of electric and thermal energy, (2) petroleum processing and coking, and (3) metals mining and dressing. Nearly 80% of CO_2_ emissions were attributed to these three sectors. On the other hand, the construction sector was the primary recipient of embodied carbon due to China’s fast urbanization, which resulted in significant infrastructure expansion. The study recommended promoting energy efficiency in manufacturing processes and reducing downstream industry usage of energy-intensive products to reduce carbon emissions.

**Table 2:** Methods used to map the direct and indirect carbon emissions. The mapping sectors, locations, used models, and data sources by different research on mapping carbon emissions are briefly described

Mapping sectors	Location	Mapping methods	References
Industrial	China	The hypothetical extraction method was used to check interdependent methods. The data used were obtained from the input–Output Table of China 2012 and the Energy Statistical Yearbook 2013	(Bai et al. 2018)
All sectors	Worldwide	The spatial estimates of emissions and economic activities were related to the standard multi-regional input–output model. Then, an extension of the monetary transaction between countries and sectors to embodied carbon emission flows was done	(Kanemoto et al. 2016)
Industry, agriculture, household, transport	Beijing, Tianjin, Hebei-China	Industrial emissions data were obtained from China industrial facility database, energy consumption data from the Chinese Energy Statistical Yearbook 2013, and transport data were calculated by authors. Then, socioeconomic data were obtained from provincial statistical yearbooks and population data from provincial population and employment statistics yearbooks. Then, the authors built a 1 km gridded spatial mapping system and used the Kaya equation for decomposition	(Cai et al. 2018)
Forests	Malawi	The data were sourced from 30 m Landsat Thematic Mapper (TM), Enhanced Thematic Mapper (ETM +), and Operational Land Imager of 2000, 2009, and 2015. Then, the authors used the fC Tool to map deforestation and forest degradation	(Skole et al. 2021)
Ecosystem	Romania	Authors created Geographic Information System maps from satellite and aero-photographs. Then biom categories associated with fauna were selected, and light detection and ranging (LiDAR) technology was used for analysis	(Mihut et al. 2019)
City	Melbourne-Australia	Authors made city maps based on environmental input–output analysis and Leontief-inverse demand-pull Input–Output calculus	(Wiedmann et al. 2016)
Forests	Venezuelan Amazon	50 Landsat 4, 5, 7, and 8-time series were used from US Geological Survey. The field data were obtained from the Industria Técnica de Maderas C.A (INTECMACA) and Empresa Nacional Forestal (ENAFOR) inventories, and reports from logging companies were used to obtain trees properties. Then, the analytical approach was done by mapping selective logging using the TerraAmazon system and validating them, then construction and validation of degradation maps, then the estimation of Aboveground Biomass and Carbon, and estimation of Committed Carbon Emissions	(Pacheco-Angulo et al. 2021)
Buildings, transportation	Sumida, Tokyo, Japan	The authors used spatial micro–Big Data, 3D carbon mapping, and a bottom-up approach model. Total emissions were estimated from Japan’s greenhouse gas Inventory Office, and unit emissions were estimated from the Japan Institute of Energy report. Then, the results were visualized in aeronautical reconnaissance coverage geographic information system (ArcGIS) 10.5	(Yamagata et al. 2018)
Urban indirect emissions	China	The authors used data from Global Change Research Data Publishing and Repository. Then used the Input–output method and logarithmic mean divisia method (LMDI-I method)	(Cui and Zhang 2018)
Industries indirect emissions	China	The authors used the Input–output analysis, carbon emissions intensity, and network theory to make the indirect carbon emissions flow network (ICEFN)	(Du et al. 2018)
Tourism direct and indirect emissions	China	The authors combined Tourism Satellite Account and the input–output model to calculate tourism industry carbon emissions. Then, the authors obtained the energy input of different industries from the China Statistical Yearbook and calculated the direct emissions of the tourism industry. Then, using input–output balance, the indirect emissions data were obtained	(Meng et al. 2016)
Household consumption indirect emissions	United States of America and China	The authors used the Input–output model. The China data were obtained from the China Statistical yearbook, and the United States of America data were obtained from the Energy Information Administration website	(Ma et al. 2016)

Another study examined the carbon emissions in the Chinese cities of Beijing, Tianjin, and Hebei. The findings indicated that per capita CO_2_ emissions in Beijing and Tianjin’s metropolitan areas were lower than provincial averages, implying that intensive human activities were recorded (Cai et al. 2018). In comparison to Beijing and Tianjin, Hebei province’s urban areas were dominated by industries with the most diverse functions. Urbanization reduced per capita CO_2_ emissions from the transportation sector, with Hebei benefiting the most. Policymakers are encouraged not to embrace a single solution but rather to impose options that consider the urban area’s breakdown.

Skole et al. used spatial and quantitative measurements to determine the rates of deforestation and forest degradation in Malawi’s forests and agricultural areas. The analysis indicated that deforestation rates between 2000–2009 and 2009–2015 were 22,410 ha yr^−1^ and 38,937 ha yr^−1^, respectively. Additionally, the forest degradation rates between 2000–2009 and 2009–2015 were 42,961 ha yr^−1^ and 71,878 ha yr^−1^, respectively. The rates revealed in this study were higher than those obtained by global forest watch since the study carried out by global forest watch considered deforestation only in government forests, excluding agricultural lands and community forests. The updated estimates are critical for developing a national policy for forest resource management (Skole et al. 2021).

Another study of the ecosystem’s carbon footprint conducted in Romania discovered a density of 2949 ha and a projected crown coverage of 7616 ha. Additionally, the forest had 27,800 m^3^ of green biomass and 13,066 t of carbon (Mihut et al. 2019). Another study on forest degradation as a result of logging was conducted in Venezuela’s Amazon (Pacheco-Angulo et al. 2021). The findings indicated that forest degradation directly impacted 24,480 ha of the Imataca forest reserve. With a harvest intensity of 2.8 ± 1.2 trees ha^−1^, selective logging released around 61 ± 21.9 MgC ha^−1^. The findings of these studies are critical for executing projects for reducing emissions from deforestation and forest degradation (REDD +).

Several researchers have attempted to map emissions by creating city carbon maps. Wiedmann et al. created a carbon footprint map for Melbourne, revealing a total of 25.1 t CO_2_-eq/capita (Wiedmann et al. 2016). Among these emissions, the industries’ emissions incorporated in local and exported products are 4.3 t CO_2_-eq/capita and 5.3 t CO_2_-eq/capita, respectively. Additionally, power generation and demand emissions totalled 10 t CO_2_-eq/capita, while import-related emissions totalled 10.8 t CO_2_-eq/capita. The primary contributors to Melbourne’s carbon footprint are households, government, and businesses, accounting for 64%, 15%, and 21% of total emissions, respectively. Here, policymakers are urged to concentrate their efforts on the social aspect of carbon emission reduction so that residents can learn how to reduce carbon emissions in their households.

Another mapping was conducted in the urban area of Sumida in Tokyo, with an emphasis on direct and indirect emissions from buildings and transportation (Yamagata et al. 2018). The study examined 46,352 and 7928 buildings and road links and discovered that road emissions were particularly high between 6:00 and 9:00 and between 15:00 and 18:00 due to intensive commuting. Emissions from buildings were particularly high between 9:00 and 18:00. Additionally, carbon emissions were highest during July compared to other months, indicating that more energy was required for cooling, necessitating increased attention, particularly as global temperatures continue to rise.

**Fig. 3:**
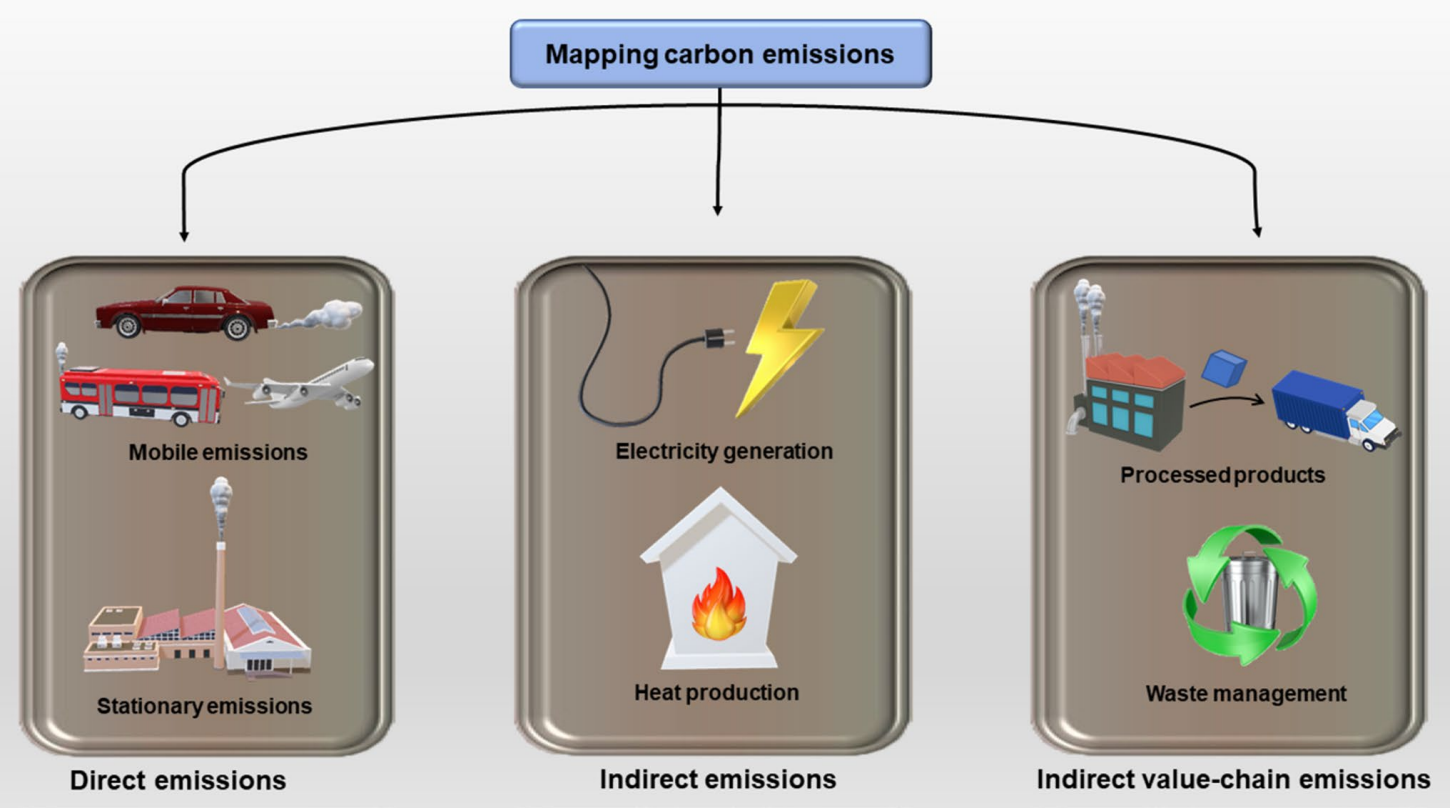
Carbon emissions are classified into three main categories, including direct, indirect and indirect value-chain emissions. Direct emissions are generated by mobile and stationary sources of direct fuel combustion. Indirect emissions are a result of the consumption of electricity or heat. Indirect value-chain emissions include those associated with the processing of products and waste management, among others

The road links had significant direct emissions from fossil fuels compared to indirect emissions, indicating that the transition from gasoline to electric vehicles will substantially reduce carbon emissions. However, the use of electric vehicles will lead to indirect emissions from electricity usage; hence more research is required in this area. The study also discovered that carbon emissions around the commercial district of Kinshi-Cho were higher than those around SkyTree due to Kinshi-Cho being unplanned and densely packed compared to SkyTree, which has well-planned energy-efficient buildings (Yamagata et al. 2018). Here, the authors propose an improvement in commuting patterns to reduce carbon emissions throughout the morning and evening hours. Additionally, a shift to more efficient and renewable energy systems will reduce carbon emissions associated with building cooling and heating systems. Figure 3 summarizes the various types of carbon emissions, both direct and indirect.

Another study examined indirect emissions across households in China and the United States of America using an input–output model (Ma et al. 2016). The findings indicated that the United States has historically emitted more indirect CO_2_ than China. However, there has been a recent trend in which China’s household emissions have increased while those in the United States of America have decreased. The trend is evident from 2000 to 2010; the United States of America have maintained indirect household emissions at 400 million tonnes while China increased from 150 to 500 million tonnes. In 2010, the United States of America’s residence; education, culture, and recreation; and transportation and communication sectors accounted for 39.5%, 15.85%, and 17.65%, respectively, of total indirect emissions. In comparison, China’s indirect emissions were accounted for by residence; education, culture, and recreation; and transportation and communication, which accounted for 50%, 2.28%, and 2.48%, respectively. Here, several policies such as government guidence to people, the development of new technology, and the promotion of energy-saving initiatives can be used to reduce emissions.

Another study estimated the direct and indirect carbon emissions produced by China’s tourism industry (Meng et al. 2016). The tourism industry generated total carbon emissions of 111.49 Mt, 141.88 Mt, 169.76 Mt, and 208.4 Mt in 2002, 2005, 2007, and 2010, respectively, accounting for 2.489%, 2.425%, 2.439%, and 2.447% carbon emissions from all industries in China. Apart from transportation, the other tourism sectors emitted three to four times the amount of direct carbon emissions indirectly. Due to the complexity of tourism carbon emissions, more research should be conducted on mapping the industry’s direct and indirect emissions.

This section discussed the various approaches used by researchers to map direct and indirect carbon emissions. The input–output model, spatial systems, geographic information system maps, light detection and ranging (LiDAR) technology, and the logarithmic mean divisia method (LMDI-I) are just a few of the methodologies and technologies used. The findings of these mapping studies assist policymakers in determining which sectors or sections of cities deserve attention, allowing for more efficient climate change policies than general approaches.

## Achieving carbon neutrality

There are generally two viable approaches explored in the literature concerning achieving carbon neutrality. The first approach entails initiatives, policies, and technologies to reduce CO_2_ emissions. In addition to emissions reductions, further measures are required to achieve a net-zero carbon system. A second approach focuses on carbon removal from the atmosphere, also referred to as negative emissions, via a variety of emerging engineered technologies and nature-based solutions.

### Carbon emissions reduction

#### Policies

Carbon emissions are reduced when low-carbon policies are implemented. Wang et al. used synthetic control and difference-in-differences methodologies to examine China’s carbon trading policies from 2008 to 2018. The findings indicated that carbon emissions decreased dramatically in several provinces following the implementation of the carbon trading policy. Additionally, the research demonstrated that the continued implementation of a carbon trading policy would result in carbon neutrality (Wang et al. 2022). Another study examined the feasibility of carbon tax incentive programmes for reducing the aviation industry’s carbon emissions using algorithms and airline data (Qiu et al. 2020). The findings indicated that, under the right circumstances, such as a low fuel price differential, incentive schemes could incentivize airline businesses to increase fuel efficiency, hence lowering carbon emissions. Carbon trading and tax policies help reduce carbon emissions and eventually lead to carbon neutrality.

Another study examined the influence of vehicle emissions policies on carbon emissions reductions by monitoring vehicle pollution in the European cities of Rome, London, and Florence using global positioning system tracing. The results indicated that particular cars and roads emit significantly more CO_2_ than others; thus, interventions such as electrification or changing travel patterns should target these large polluters rather than enacting broad carbon emission policies (Böhm et al. 2021). Low carbon policies are critical for lowering carbon emissions; yet, policymakers should examine the economies of their particular communities to ensure that economic development is not adversely affected. Overall, the role of policies implemented towards carbon emissions reduction such as carbon trading, carbon tax and targeted policies is critical and requires careful examination by policymakers.

#### Sector specific technologies and initiatives

Energy-related emissions are the primary contributor to rising greenhouse gas concentrations in the atmosphere; hence, typical emission reduction strategies and efforts should target both the energy supply and demand sides. The literature generally discusses emission reduction efforts regarding technologies and strategies used in four primary sectors: power on the supply side and industrial, buildings and transportation, on the demand side. Emission reduction can be accomplished within the power sector by introducing renewable energy, carbon capture and storage, nuclear power, and supply-side fuel switching to low-carbon fuels. Additionally, demand-side emission reduction efforts include efficiency gains realized through the implementation of energy-efficient processes and sector-specific technologies that reduce energy consumption, as well as end-use fuel switching from fossil-based to renewable fuels and the deployment of renewable energy technologies (Fawzy et al. 2020). Another important sector contributing to carbon emissions is agriculture and animal farming.

##### Energy

Investment in clean energy and energy efficiency are critical elements of reducing carbon emissions. Juan et al. examined the impact of globalization and renewable energy on Brazil, India, China, and South Africa’s carbon neutrality targets (Juan et al. 2021). The study examined economic and energy indicators from 1980 to 2018 utilizing statistical models such as fixed effect and random effect models. The findings indicated that increasing globalization by 1% increases carbon emissions by 0.0342%, whereas increasing a unit of renewable energy consumption such as wind and hydropower reduces carbon emissions by 0.0143%. These findings demonstrate that renewable energy sources are an efficient way to reduce carbon emissions.

Based on carbon footprint measurements, another study conducted in Bangladesh quantified the environmental implications of energy consumption from 1975 to 2016. The findings indicated that increasing per capita hydroelectricity consumption by 1% reduced the carbon footprint by 0.02–0.03%, all other things being equal. Renewable energy sources improve environmental quality; nevertheless, their use in Bangladesh for electricity production is as low as 1%, which is insufficient to reduce carbon emissions (Murshed et al. 2021). Another study conducted in China found that wind and solar capacity of 2495 and 2674 GW can meet 67% of China’s total energy consumption in 2050, respectively (Liu et al. 2022a). Additionally, the analysis revealed that supplying 10.4 PWh of renewable energy annually would result in a reduction of 2.08 Mt SO_2_ and 1.97 Mt NO_x_, bringing the country closer to meeting its carbon neutrality targets. In summary, achieving carbon neutrality is not a one-day accomplishment. Long-term strategies that promote renewable energy and energy efficiency are necessary to reduce carbon emissions and attain carbon neutrality.

A transition away from fossil fuel energy sources and toward renewable energy sources is critical to attaining future carbon neutrality. Millot and Maïzi examined previous energy transitions and discovered that they occurred spontaneously as a result of technology advancements, economic, social, and political benefits such as lower prices and increased living comfort (Millot and Maïzi 2021). On the other hand, the shift to carbon neutrality cannot occur spontaneously because carbon neutrality is primarily motivated by environmental concerns and offers no immediate financial benefits to individuals or corporations. As a result, low-carbon technologies must be competitive in order to facilitate this energy transition. Additionally, governments should work to price carbon, support research and development, and advance technological innovation.

Another study was conducted on future energy systems in Europe in order to achieve carbon neutrality by mid-century utilizing the European TIMES model (ETM-UCL), price-induced market equilibrium system (PRIMES), and regional model of investments and development (REMIND) energy environment-economy models (Rodrigues et al. 2022). The findings indicated that carbon neutrality is technically feasible with future energy technologies by mid-century. The energy transition solutions proposed include electrifying energy services such as vehicles and heat pumps, altering lifestyles, improving energy efficiency, and promoting renewable energy. Because renewable energy is critical to reaching carbon neutrality, governments, financiers, legislators, and academics should make renewable energy a major priority (Fawzy et al. 2020). Schiffer and Trüby examined Germany’s energy strategy, dubbed the “Energiewende”, which was implemented in 2010 with the goal of achieving carbon neutrality in the country. Until 2018, the energy programme was well-executed but fell short of reducing CO_2_ emissions. A recommendation is that the energy transformation should begin with electricity generation and expand to the transportation, industrial, and building sectors to cut emissions (Schiffer and Trüby 2018). Additionally, international cooperation should be fostered to achieve global carbon neutrality targets.

Overall, the impact of transitioning from fossil-based to renewable energy is well documented and is considered the most important approach to achieving carbon neutrality in the energy sector. However, it is important to note that such transition cannot happen spontaneously. Governments, financiers, legislators, and academics need to focus on promoting renewable energy systems.

##### Industry

In relation to the industrial sector, Griffin and Hammond researched the carbon emissions reduction for the iron and steel industry in the United Kingdom, accounting for 26% of the total industry-related greenhouse gas emissions in the country. The blast furnace was the most efficient and the highest energy user of all the steel production processes, requiring attention to achieve carbon neutrality. The research recommended energy-saving technologies such as heat recovery in coke ovens, sinter plants, and electric arc furnaces. The utilization of such technologies results in an 18% reduction in energy consumption and a 12% reduction in greenhouse gas emissions. Additionally, the study concluded that carbon emissions reductions until 2050 are possible with the use of efficient production processes and a shift to bioenergy (Griffin and Hammond 2019).

Using an environmental-economic simulation model, another study examined the carbon emission reductions of China’s iron and steel industry by 2030 (Li et al. 2019). The simulation considered many scenarios, including business, as usual, industrial upgrading, carbon taxation, carbon trading, and a combination of all scenarios. Carbon emissions were well controlled in the industrial upgrade’s scenario, while carbon taxes encouraged low emissions technologies. Furthermore, a combination of all scenarios resulted in the most effective carbon emission reductions, meeting China’s target. In summary, the iron and steel industries’ key to emissions reductions is the use of sustainable technical processes and energy sources. Furthermore, Arens et al. examined worldwide steel production and its transition away from coal-fired power generation, as coal-fired steel manufacturing currently accounts for 8% of global energy CO_2_ emissions. According to the analysis, the steel industry is not well-positioned to achieve carbon neutrality by 2050. Except for members of the European Union, other countries have not demonstrated a strong commitment to energy transition and decarbonization (Arens et al. 2021).

Another study examined the glass manufacturing sector and its decarbonization process, noting that the container and flat glass industries alone release over 60 million tonnes of CO_2_ per year and that around 75–85% of energy is used to heat raw materials in a furnace (Furszyfer Del Rio et al. 2022). Carbon capture and storage, batch preheating, biofuels, electric furnaces, technical heating and melting, and glass waste recycling were all addressed as approaches to create a low carbon glass industry. Additionally, the study identified impediments to the glass industry’s decarbonization, including a shortage of capital, fluctuating energy prices, and unreliable infrastructure.

The forestry industry contributes a substantial amount of CO_2_ to the atmosphere, requiring attention. The forestry industry in Finland and Sweden was studied by identifying the sectors with the highest emissions, including transportation, non-road machinery, lime kilns and dryers, onsite energy production, and purchasing power (Lipiäinen et al. 2022). Several techniques for decarbonization have been proposed, including switching to biofuels for energy and electrifying the forestry industry’s transportation sector. However, effective regulations and incentives are essential to accomplish a realistic level of decarbonization while avoiding negative consequences. For example, excessive demand for biofuels can lead to over-demand in biomass, increasing price and scarcity.

Besides the obvious benefits of fuel-switching and the use of renewable energy technologies, there are many opportunities for industrial operations to benefit from efficiency improvements in order to reduce carbon emissions. In the steel and cement sectors, waste heat from exhaust gases can be used for onsite power and heat production via waste-heat driven power plants. In process industries that use steam, there are various opportunities for efficiency gains, starting from efficiency improvements that are carried out in the boiler, followed by the installation of back pressure turbines in areas where pressure reduction is required to generate additional electricity. Furthermore, energy efficiency improvements can be realized by deploying advanced equipment control systems across a multitude of industries (Fawzy et al. 2020).

Overall, there are various approaches to reduce carbon emissions in the industrial sector. This includes fuel-switching from fossil-based to renewable fuels and the deployment of various technologies to promote energy efficiency. Furthermore, the re-utilization of waste energy sources and the introduction of renewable energy systems into the energy-mix of such industrial processes are promising approaches. These measures can be implemented across a wide range of industries.

##### New carbon emission reduction technologies

Furthermore, carbon capture, utilization and storage (CCUS) is emerging as a promising technology that has been addressed in the literature as a possible strategy to reduce emissions in both the power and industrial sectors. The method entails separating and capturing CO_2_ gases produced by processes that utilize fossil fuels. The captured CO_2_ is then transported and stored for very long periods of time in geological reserves. Alternatively, the captured CO_2_ can be used to produce chemicals, algae, and concrete building materials, as well as being used in enhanced oil recovery. The primary objective is to reduce emissions while continuing to use fossil fuels. The literature discusses three capture technologies: pre-combustion, post-combustion, and oxyfuel combustion. Each technique has a distinct process for CO_2_ extraction and capture. However, post-combustion capture systems are ideal for retrofit projects and have a wide range of applications (Fawzy et al. 2020; Osman et al. 2020).

Furthermore, advancements in capture technologies are required to enhance efficiency and consequently improve the costs of such systems. Lei et al. reviewed the application of carbon membrane systems in different processes such as hydrogen purification, capturing CO_2_ during combustion, and natural gas sweetening. For CO_2_ capture, carbon membranes have advantages such as low energy consumption and footprint compared to other CO_2_ capture methods like amine absorption. The carbon molecular sieve membrane has a high separation performance of CO_2_/NO_2_ with a 2140 Barrer permeability of CO_2_ even at high humidity (~ 90%). However, the use of carbon molecular sieve membranes in flue gas separation has drawbacks, such as the huge area required to capture a given amount of CO_2_ and deteriorating performance over time due to carbon matrix species sorption. The authors recommended the development of ultra-thin carbon molecular sieve membranes that are highly hydrophobic (Lei et al. 2020).

In conclusion, carbon capture, utilization and storage, where carbon captured can be stored or utilized in production of chemicals, algae, and concrete building materials is an emerging technology that can play a pivotal role in achieving carbon emission reductions. However, it should not be a solution that encourages the continued use of fossil-based energy.

##### Buildings and cities

Due to the increasing urban population and the amount of time people spend in buildings, buildings and cities are responsible for significant amounts of carbon emissions that contribute to climate change. For cities, one strategy for adapting to climate change is to develop resilient designs capable of withstanding natural disasters while minimizing the impact on the natural environment (Wang et al. 2018). Additionally, mitigation can be attained by deploying decentralized energy systems for cities; however, this option has a significant initial cost.

Buildings can achieve a carbon-free future by utilizing improved building envelopes, renewable materials, and 3D printing. Additionally, this can be achieved by developing heating and cooling systems powered by renewable energy and employing energy-efficient technologies (Fawzy et al. 2020). Furthermore, the use of sensors to monitor and regulate smart building equipment such as lighting, as well as the development of electric and thermal energy storage systems, are promising approaches. Moreover, electromechanical equipment in buildings should be eco-labelled, and minimum standards for heating, ventilation, and air conditioning systems should be implemented.

The reintroduction of lumber into structures is critical because a cubic metre of wood stores half a tonne of carbon; hence, timber buildings and cities can act as carbon sinks. Additionally, combining and covering construction materials with nanoparticles improves their characteristics, increasing their sustainability. In summary, significant work must be done on new construction and retrofitting existing structures to align with carbon neutrality programmes and objectives. However, careful planning is essential to avoid poor and optimistic plans that result in unreachable goals, such as the failed smart cities initiatives in several countries.

Overall, buildings and cities play a critical role in reducing carbon emissions and achieving carbon neutrality. Several strategies are suggested, including resilient designs, decentralized energy systems, improved building envelopes, renewable energies, eco-labelling and the use of lumber in construction.

##### Transportation

The transition of the transportation sector to renewable energy is challenging, particularly for large, long-range vehicles and aircraft (Dominković et al. 2018). Several alternatives to fossil fuels have been proposed, including biofuels, hydrogen, electro-fuels, and electricity. Electricity offers the greatest number of benefits, including higher efficiency, reduced CO_2_ emissions, and improved air quality in the transportation sector. For instance, electricity can provide 72.3% of the total energy necessary for transport in the European Union using existing technologies.

Another study examined the life expectancy of electric vehicle batteries in the context of a circular and low-carbon economy (Bonsu 2020). The study identified several issues associated with electric car batteries, including ethical concerns, excessive extraction of raw materials for batteries, a lack of policies addressing manufacturing emissions, and a lack of research and a market for end-of-life batteries. The analysis demonstrated that a circular economy can achieve net-zero carbon emissions by 2050. However, the circular economy should not be limited to recycling raw materials and repurposing batteries; circular economy should also consider issues such as equitable employment, value chain emissions, environmental protection, and responsible natural resource consumption.

Wu et al. investigated the obstacles and solutions to the deployment of hydrogen fuel cell vehicles in China, identifying barriers such as insufficient supporting infrastructure, a scarcity of manufacturers, and concerns about hydrogen fuel safety. To accelerate the transition to carbon-neutral transportation, the study recommended developing hydrogen supply chains, ensuring the safety of hydrogen supplies, and expanding financial support and research (Wu et al. 2021). Another study examined the use and potential of biogas in transportation in the European Union by upgrading biogas to biomethane (Prussi et al. 2019). By 2030, the usage of biomethane in vehicles for compressed natural gas and liquified natural gas will increase to 30 billion m3/yr. Additionally, biomethane will be used in maritime and inland waterway transportation. In summary, decarbonizing the transportation sector is feasible through the use of electricity, biofuels, hydrogen, and electro-fuels, with electricity being the most practical alternative. However, adequate research should be conducted on the proper disposal of end-of-life batteries in order to ensure sustainable energy for the transportation sector’s entire lifecycle.

Other forms of efficiency measures are viable in the transportation sector. The introduction of travel demand management to reduce travel frequency and distance may also contribute to efficiency improvements in the transportation sector (Fawzy et al. 2020). Furthermore, the growth of sharing economies, such as sharing rides, parking spaces, and crowdsourcing information, would increase the sector’s efficiency, resulting in decreased carbon emissions.

In conclusion, the electrification of the transportation sector was found to be the best way to lower the sector’s carbon emissions. Other strategies to reduce carbon emissions in the transportation sector include electro-fuels, hydrogen, biofuels, as well as other efficiency measures such as travel demand management and the promotion of sharing economies.

##### Agriculture, food, and waste

Agricultural land use, food consumption habits, and waste disposal all contribute significantly to greenhouse gas reduction. Strapasson et al. used the European Union land-use futures (EULUF) model to examine the effect of food consumption and agricultural practices on greenhouse gas emissions in the European Union. The study concluded that shifting to a more vegetarian diet, consuming less meat, and reducing food waste will mitigate climate change. Additionally, increased livestock yields and soil carbon in pasture lands minimize the livestock sector’s carbon impact (Strapasson et al. 2020).

Another study in South America examined the possibilities for low-carbon agriculture to help reduce climate change and promote food security. South America accounts for 31.3% of global annual greenhouse gas emissions from land use and land-use change, according to the study (Sa et al. 2017). Between 2016 and 2050, South America’s potential as a carbon sink through low-carbon agriculture was 8.24 PgC. Agriculture’s contribution to climate change mitigation was estimated to be 31% through pasture restoration, 25.6% through the crop, livestock, and forestry integration, 24.3% through no-till farming, 12.8% through forestation, 4.2% through biological nitrogen fixation, and 2% through industrial organic waste recycling. Additionally, low carbon agriculture can improve food and meat output by 17.6 Mt.year^−1^ and 1.6 Mt.year^−1^, respectively. A recommendation to policymakers is to devise means of incentivizing the public to adopt sustainable land-use practices and healthy diets.

Global population growth results in a rise in agricultural and food waste. Incineration and landfilling both have drawbacks in terms of greenhouse gas emissions and environmental pollution. Rao and Rathod investigated various methods for repurposing food and agricultural waste in order to attain carbon neutrality. Food and agro-waste can be used to produce new pharmaceuticals, phytochemicals, enzyme immobilization, heavy metal removal from wastewater, and waste cooking oil that can be converted to biodiesel. The study concluded that while these applications have been investigated in the laboratory, they should be scaled up to realize their benefits (Rao and Rathod 2019). In summary, adopting low carbon agriculture, changing eating behaviours, and valorizing food and agro-waste implementation is essential to achieving a carbon-free future.

Overall, the main strategies to reduce carbon emissions around agriculture, food and waste include shifting to vegetarian diets, reducing food waste, pasture restoration, no-till farming, and repurposing food and agricultural wastes.

#### General societal initiatives

Apart from corporations and governments, individuals and households are critical in reducing carbon emissions. Pulselli et al. quantified greenhouse gas emissions from households in European cities and examined mitigation strategies. A typical household’s carbon footprint was determined to be 6.93 t CO_2_-eq/yr, which corresponds to the annual carbon absorbed by 0.51 hectare of forest (Pulselli et al. 2019).

Another study examined the carbon footprints of households in Berlin, Germany, comparing voluntary carbon emission reductions in 2018 to involuntary carbon emission reductions during the coronavirus disease 2019. Carbon trackers were installed in the households to monitor their carbon footprints associated with electricity use, mobility, and food intake. The findings indicated that households saved an average of 11% in carbon emissions, with some people saving up to 40% (Reusswig et al. 2021). The households highlighted various difficulties in reducing emissions, such as concerns about road safety, which prevented them from converting to bicycles. The emergence of the coronavirus disease 2019 resulted in a 10% reduction in carbon emissions in Germany, but scientists expected that emissions would increase when economies recovered following the coronavirus disease 2019. Households can implement several low-cost mitigation strategies to help reduce carbon emissions, including shading facades, efficient lighting use, walking or cycling to work, carpooling, and public transportation use.

Apart from households, universities can help reduce carbon emissions. Carbon emissions were quantified at the NED University of engineering and technology in Karachi, Pakistan, using a carbon calculator, and mitigating strategies were identified (Mustafa et al. 2022). The data indicated that the campus produced 21,500 Mt CO_2_-eq in 2017, equating to 1.79 Mt CO_2_-eq per student. The key mitigation methods suggested were the adoption of renewable energy sources, the use of energy-efficient appliances, the conversion to electric vehicles, and the planting of trees. Thus, because households and individuals are critical components of achieving carbon neutrality, climate change education should be provided to educate individuals about strategies to minimize carbon emissions at home, school, and work.

As noted, society can play an important role in carbon emission reduction. The suggested strategies include shading facades, efficient lighting use, walking or cycling to work, energy-efficient appliances, converting to electric vehicles, planting of trees, and climate change education.

### Atmospheric carbon removal

Carbon neutrality cannot be achieved solely through carbon emissions reduction; therefore, negative emissions technologies are required to attain a carbon-free world. The primary negative emissions techniques that have been extensively discussed in the literature include bioenergy carbon capture and storage, direct air carbon capture and storage, biochar, soil carbon sequestration (Fawzy et al. 2020). This is along with afforestation and reforestation, enhanced terrestrial weathering, wetland construction and restoration, ocean alkalinity enhancement, and ocean fertilization, as well as alternative storage approaches such as mineral carbonation and the use of biomass in construction (Fawzy et al. 2020). Each of these techniques carries its costs, challenges, limitations and merits.

In Scotland, a study was conducted to determine the energy and economic costs associated with adopting land-based negative emissions technologies (Alcalde et al. 2018). Bioenergy carbon capture and storage, direct air capture, enhanced weathering, forest sink capacity, soil carbon sequestration, and biomass conversion to biochar are the technologies investigated. Economically, the enhanced weathering approach had the highest costs, with lower and upper costs of $US 25/t CO_2_ and $US 1600/t CO_2_, respectively. On the other hand, bioenergy carbon capture and storage and forestation were less expensive, whereas biochar and soil carbon sequestration could be cost-effective. The study advised implementing a mix of bioenergy carbon capture and storage, soil carbon sequestration, and enhanced weathering technologies, which has the potential to reduce emissions by 8.3–36.8 Mt CO_2_. The combined maximum capacity could eliminate up to 89.8% of Scotland’s annual emissions. In addition, bioenergy can be produced through thermochemical processes which are more efficient in time and conversion rate or biochemical processes which produce more volatile organic compounds and require less energy and temperature (Liu et al. 2022b). When compared to a single negative emissions technology, a combination of different negative emissions technologies that act in concert produces the best results with the least amount of resource use.

Fuhrman et al. investigated the negative emissions technologies’ impacts on food, energy, and water resources. According to the study, direct air carbon capture technology can achieve negative emissions of 3 Gt CO_2_yr^−1^ by 2035 at current pricing and efficiency levels. Additionally, direct air carbon capture avoids the land use demand and food crop crisis difficulties associated with bioenergy carbon capture and storage and afforestation. The study concluded that policymakers considering negative emission technologies should take into account non-climate-related environmental implications (Fuhrman et al. 2020).

Negative emission technologies, on the other hand, have some drawbacks. Utilizing the impulse response function, a study investigated the risk of carbon leakage into the atmosphere as a result of using negative emissions technologies. The results indicated that over various time scales of leakage and assuming that 80% carbon was permanently stored, the leakage to the environment was negligible at 3 parts per million CO_2_ (Lyngfelt et al. 2019). In conclusion, leakage is unlikely to have a substantial negative impact on the accomplishment of carbon-negative emissions unless an excessive amount of leakage occurs.

Another study concluded that negative emissions technologies are not yet ready for widespread use due to uncertainty regarding their technologies, pricing, and environmental implications (Chavez 2018). Thus, the research concluded that measures such as renewable portfolio standards, which require electricity companies to offer a specific percentage of their energy supply using eligible renewable sources, should be established to expedite the development of negative emissions technologies. Applying a similar policy in carbon removal will drive investment in negative emissions technologies. In summary, negative emissions technologies have enormous promise for achieving future carbon neutrality; hence, additional investment, research, and regulations are needed to encourage deployment.

In conclusion, negative emissions technologies can contribute to achieving carbon neutrality. However, each of these technologies requires a different level of investment, operating conditions, and energy demand, which implementers should consider when scaling them up. Additionally, researchers should conduct a comprehensive life cycle analysis of these technologies to ensure they are implemented efficiently and at the lowest possible cost.

## Life cycle analysis of various carbon neutral systems

A life cycle analysis is a technique for determining the environmental impact of a product system across its useful life (Finnveden et al. 2009; Rebitzer et al. 2004). The life cycle analysis process begins with formulating objectives, and the scope for a life cycle inventory continues with a life cycle impact analysis and concludes with the interpretation and translation of the results (Corporation and Curran 2006). Life cycle analysis is also frequently utilized in various carbon-neutral systems to characterize greenhouse gases, and related climate change impacts objectively (Osman et al. 2021). For example, Wiloso et al. investigated the impact of biochar inventories on bioenergy life cycle analysis (Wiloso et al. 2016), Petrovic et al. explored the life cycle analysis of building materials for single-family houses in Sweden (Petrovic et al. 2019), and Thonemann and Pizzol analysed the corresponding carbon capture and utilization technologies in the chemical industry (Thonemann and Pizzol 2019). As some nations have established targets to achieve carbon neutrality, life cycle analysis has been used to assess biological, building, materials, chemical, and other carbon-neutral systems. Table 3 summarizes various carbon-neutral systems in different countries and sectors that have used life cycle analysis.

**Table 3:** Life cycle analysis of various carbon neutral systems. Table 3 investigates different countries that have adopted life-cycle assessment methods in carbon-neutral systems in different areas

Sector	Project description	Country	Year	Key findings	Reference
Transportation	Transport carbon modelling in the United Kingdom: an integrated life cycle approach to exploring a low carbon future	The United Kingdom	2012	This study presents the United Kingdom Transport Carbon Model, which can develop transport policy scenarios that explore the full range of technical, fiscal, regulatory, and behavioural change policy interventions to achieve the United Kingdom’s climate change and energy security goals	(Brand et al. 2012)
Forestry	A critical analysis of carbon-neutral assumptions in life cycle assessment of forest bioenergy systems	China	2017	This study critically analyses the carbon neutrality assumptions in the life cycle assessment model for assessing the climate change impacts of bioenergy use such that the climate change impacts of bioenergy use can be accurately assessed	(Liu et al. 2017)
Building	Smart windows for carbon-neutral buildings: a life cycle approach	Italy	2018	The study evaluated the life cycle impact of photocell windows on office buildings and total life cycle energy consumption. Its smart windows have proven beneficial technology and a possible solution for commercial buildings to meet near-zero energy building and carbon-neutral building standards	(Pierucci et al. 2018)
Biology	Key issues and options for accounting for carbon sequestration and interim storage in life cycle assessment and carbon footprinting	Italy	2013	This paper reviews and discusses six existing methods for accounting for the potential climate impacts of carbon sequestration and temporary storage or release of biogenic carbon in life cycle assessment and carbon footprinting	(Brandão et al. 2013)
Drainage	Life cycle assessment of water and wastewater systems in Trondheim, Norway: a case study	Norway	2014	This study presents the results of a life cycle assessment of the water and wastewater systems in the city of Trondheim. The study results were used to plan a new carbon-neutral urban settlement	(Slagstad and Brattebø 2014)
Chemistry	Sustainable conversion of carbon dioxide: an integrated review of catalysis and life cycle assessment	Germany	2018	This paper assesses the potential for reducing the environmental footprint in these applications relative to the current petrochemical value chain. The paper also mentions that advances in synthetic methods with CO_2_ as an essential component present a challenge for long-term assessment methods to provide a sound and comprehensive assessment of environmental impacts	(Artz et al. 2018)
Biology	The impact of biochar inventories on bioenergy life cycle assessment: a challenge to the neutrality assumption	Netherlands	2016	This paper analyses eight scenarios focusing on various carbon flows, including biomass decomposition in the field and alternative uses as a bioenergy feedstock, regarding general bioenergy systems to coordinate future bioenergy life cycle assessments	(Wiloso et al. 2016)
Material	Life cycle assessment of building materials for single-family houses in Sweden	Sweden	2019	The life cycle assessment results in this study demonstrate the environmental impacts associated with building materials from the production and construction phases, including transportation, replacement, and deconstruction phases	(Petrovic et al. 2019)
Chemistry	Corresponding life cycle assessment of carbon capture and utilization technologies in the chemical industry	Germany	2019	The study evaluated 12 CO_2_ conversion technologies to provide decision support for each technology’s potential life-cycle environmental impacts to better achieve carbon neutrality in the introduction of carbon capture and utilization technologies in the chemical industry	(Thonemann and Pizzol 2019)

Carbon neutrality expresses a state in which individuals, products, and the activities of countries, cities, companies, and other organizations strive to emit zero carbon dioxide. Net-zero emissions indicate that their activities do not release greenhouse gases or use other technical means to decarbonize emissions or remove atmospheric carbon. While decarbonization plans are one method of achieving carbon neutrality, all decarbonization techniques must undergo a life cycle evaluation to avoid greenwashing. Additionally, carbon removal projects need to be examined from a life cycle perspective.

According to Table 3, which details the adoption of life cycle assessment studies in various carbon neutral systems across different countries and sectors, we discovered that, while the majority of countries signed the Paris agreement in 2015 to achieve carbon neutrality, researchers in the United Kingdom, Norway, and Italy had already adopted life cycle assessment in 2012, 2013 and 2014 in the transportation, biology, and drainage sectors, respectively, to achieve carbon neutrality. Additionally, between 2017 and 2019, researchers from China, Italy, Germany, and Sweden investigated studies that combined life cycle assessment methods with carbon-neutral systems in the domains of forestry, architecture, chemistry, and materials science. The majority of these studies evaluated a model, technology, or material’s ability to achieve carbon neutrality across its entire life cycle. Additionally, carbon neutrality is a twenty-first-century trend that can integrate a life cycle perspective into organizational and decision-making environments, although pure life cycle assessments have not yet accomplished this goal (Finkbeiner and Bach 2021).

In conclusion, we identified that most projects use life cycle assessment to analyse carbon neutrality technologies or models and use a life cycle perspective to incorporate organizational and decision-making environments. The life cycle analysis of entire systems should be enhanced and detailed from the cradle to the grave in order to ensure overall system carbon neutrality.

## Sustainability resulting from carbon neutrality

Carbon neutrality is a new industrial revolution that humanity faces, one that will progress toward a carbon-free and sustainable future, which will have a major effect on the environment, society, and economy (Fawzy et al. 2021).

### Impact on the environment

The ultimate goal of the Paris climate agreement is to keep global warming below 2 ℃ and try to limit it to below 1.5 ℃ (Rogelj et al. 2016). One of the most severe environmental problems currently facing the world is climate change. The overexploitation of non-renewable resources by global industrial development and other forms of environmental damage such as heavy reliance on fossil fuels, deforestation, and waste incineration have resulted in an increase in greenhouse gases in the atmosphere, ultimately causing environmental degradation such as global temperature increase and melting of the north and south pole glaciers (Tan and Wang 2021). The primary and most direct benefit of achieving carbon neutrality is to mitigate negative environmental impacts and slow the rising rate of global temperature. As a result, achieving carbon neutrality is a critical objective for a variety of countries today and one of the possible solutions to the problem of climate change (Udemba 2021).

Since each country faces unique environmental challenges, its steps vary, but they all eventually aim to reduce negative environmental impacts and attain carbon neutrality. As a result, on the path to carbon neutrality, we can encourage the development of various measures, including those addressed in this review. Simultaneously, achieving carbon neutrality will help decrease global warming and resolve the world’s energy dilemma while also having good ecological impacts such as improved air quality, more sustainable landscapes, and ecological restoration (Chen 2021). Carbon neutrality is critical for humanity to coexist in harmony with nature and progress toward a future sustainable environment.

### Impact on society

If the world does not implement a series of measures to control global warming, human beings will confront environmental deterioration, including increased global temperatures, more frequent extreme weather, and significant harm to land and marine ecosystems (Zou et al. 2021). If global temperatures rise by 2 °C, around 13% of terrestrial ecosystems will be destroyed, and many animals and plants will become extinct; sea levels will rise by approximately 36 to 87 cm, and approximately 95% of coral reefs will face extinction (IPCC 2018). Achieving carbon neutrality would decrease the frequency of catastrophic disasters, and its direct impact on society would be to preserve the existing social order, while its indirect impact would be to promote human society’s evolution.

Extreme weather frequently results in house collapses, human casualties, and crop failures, resulting in habitat destruction, loss of loved ones, and food scarcity, severely affecting the existing social order. Additionally, the vast number of trees that felled would dramatically reduce forest cover, and many wild creatures will lose habitat, expediting species extinction and severely damaging the biological chain. The collapse of the biological chain will destroy the ecosystem, eventually resulting in human beings becoming unable to survive and the destabilization of society. Additionally, rising sea levels will result in the global inundation of some island countries and coastal towns, resulting in enormous human migration and potentially wars, threatening global social stability. As countries take various steps toward carbon neutrality, humanity is confronted with new technologies and measures that contribute to the progress of society at large.

### Impact on the economy

Several of the initiatives adopted to attain carbon neutrality will have a substantial economic impact (Ji et al. 2021). The economic impact of carbon neutrality is mostly due to a shift in economic development models, as well as energy production and consumption. Carbon neutrality will reorient economic growth toward green, low-carbon, and sustainable development; it will also significantly impact emerging technology trends, such as decarbonization technologies, energy efficiency technologies, recycling technologies, and new power systems energy storage technologies, as well as negative emissions technologies. Additionally, new models will almost certainly displace certain economic activities or businesses; for example, the existing coal industry and its accompanying infrastructure, manufacturing, and service sectors will likely continue to lose jobs. On the other hand, carbon neutrality will spur job creation in the clean energy, carbon-free energy, and renewable energy sectors, resulting in economic shocks.

Continued advancement of carbon neutrality targets is projected to considerably impact the development and reorganization of the energy mix, particularly in the higher carbon-emitting oil, coal, and natural gas sectors. Consumption of refined oil will gradually be phased out in the oil sector; countries with a high demand for crude oil consumption will see their consumption steady and then fall. The coal sector’s backward production capacity will progressively be phased out; the coal chemical industry, coal power, and other coal conversion industries will face limited expansion space; and alternative energy sources will steadily weaken and eventually replace coal use. The gradual use of alternative energy sources will gradually reduce the demand for natural gas in the energy sector. Meanwhile, in the field of non-fossil energy, new energy technologies will accelerate their development on a wide scale, and the development of its new power system will continue, gradually building a new energy consumption economy.

In conclusion, the positive impacts of carbon neutrality on the environment, society and the economy are clear. Carbon neutrality makes a significant contribution to reversing the environmental degradation that has occurred in recent years and to promoting the development of a sustainable environment for future generations. In terms of society, reaching carbon neutrality contributes to the development of a stable society, social growth, and the creation of new technologies and measures. Finally, achieving carbon neutrality will encourage a shift in economic development models, energy production and consumption, and the eventual emergence of a new economic system based on energy consumption.

## Conclusion

This comprehensive assessment of the literature examined the critical nature of achieving net-zero carbon emissions in order to support sustainable development. It began with a systematic review of the 26th United Nations Climate Change Conference of the Parties, a once-in-a-generation opportunity to reduce the adverse impacts of climate change and achieve carbon neutrality in the aftermath of the coronavirus disease 2019 pandemic. Simultaneously, the four outcome targets presented at the 26th United Nations Climate Change Conference of the Parties were studied further. The results of the four targets have far-reaching positive implications for achieving carbon neutrality globally. Meanwhile, this study gave a full and exhaustive overview of worldwide initiatives to attain carbon neutrality, the majority of which are policies or measures implemented by specific countries. Only 4.5% of the 198 countries examined have reached carbon neutrality, while most of the remaining countries are still planning to do so, with the majority of them aiming for carbon neutrality after 2050.

Additionally, this research systematically examined the interconnections and synergies between adaptation and mitigation strategies and their associated benefits. Certain strategies found in the analysis may have a detrimental effect on the objective of net-zero carbon emissions. The investigation indicated that synergies across countries are lagging. Only a quarter of Climate Change and Political Stability in Europe incorporate an in-depth investigation of mitigation and adaptation synergies. It is worth noting that the various methods for mapping direct and indirect carbon emissions (input–output models, spatial systems, geographic information system maps, LiDAR techniques, and LMDI-I methods), as well as systematic survey analysis, can be extremely beneficial for decision-makers in determining precisely which urban areas should be concerned about in order to develop more effective and targeted climate change policies.

In addition, this assessment included several sustainable strategies for achieving carbon neutrality in various sectors. The first step is to shift away from fossil fuel energy and toward renewable sources of energy, as well as to develop low-carbon technologies. The strategy for energy transition should be to electrify energy services, increase energy efficiency, and promote renewable energy sources. Simultaneously, modifying dietary habits (more vegetarian, less meat, less food waste) can help minimize the negative effects of climate change in terms of agricultural land use, food consumption patterns, and waste disposal. Pasture restoration, integrated crop-livestock-forestry systems, no-till agriculture, afforestation, biological nitrogen fixation, and organic waste recycling are all examples of climate change mitigation techniques. Adopting low-carbon agriculture, altering consumer behaviour, and raising the value of food and agricultural waste are essential steps toward net-zero carbon emissions.

In terms of buildings and cities, buildings should be designed to be resilient to natural hazards while minimizing disruption to the natural environment. Cities with decentralized energy systems and technology such as electric vehicles, the Internet of things, and big data can significantly contribute to climate change mitigation. Much effort has to be made to construct and adapt existing buildings to meet carbon-neutral goals and ambitions. On the industrial front, encouraging enterprises to switch to biofuels as an energy source via proper legislation and incentives is a sound strategy. Carbon neutrality for the industry in the future can be reached by climate and energy legislation, non-hydro, non-biofuel renewable energy expansion, and the circular economy, as well as the deployment of new technologies such as carbon capture, utilization and storage. Transitioning the transportation sector to clean energy is a challenging task, and establishing a hydrogen supply chain for new vehicles, ensuring secure hydrogen supply management, and increasing financial support is definite approaches to promote environmental change. The application of negative emissions technologies, as well as for biotechnology, has the potential to create a carbon-free planet in the future, and additional investment, research, and legislation promoting negative emissions solutions are required.

Furthermore, life cycle assessments are necessary for all decarbonization measures as well as other aspects of carbon-neutral systems. In the future, life cycle analysis should be enhanced to determine how to achieve system-wide carbon neutrality. Achieving net-zero carbon emissions can benefit the environment, society, and economy, hence promoting global sustainable development.

## Abbreviations

COP26: The 26th United Nations Climate Change Conference of the Parties
LiDAR: Light detection and ranging
LMDI-I: Logarithmic mean divisia index
CCUS: Carbon capture utilization and storage
BECCS: Bioenergy and carbon capture and storage
CO_2_: Carbon dioxide
SO_2_: Sulphur dioxide
NO*x*: Nitrogen oxides
NSPG: North and South Pole Glaciers
UNFCCC: United Nations Framework Convention on Climate Change
PCCC: Paris Climate Change Conference
IPCC: Intergovernmental Panel on Climate Change
ICEFN: Indirect carbon emissions flow network
WHO: World Health Organization
EULUF: European Union using the European Union Land Use Futures
N/A: Not available
URL: Uniform resource locator
ArcGIS: Aeronautical reconnaissance coverage geographic information system
SCPRC: State Council of the People's Republic of China
ha: Hectare
ha^−1^: Per hectare
yr^−1^: Per year
m^3^: Cubic meter
t: Tonne
Mg: Megagram
Mt: Megatonne
Gt: Gigatonne
GW: Gigawatt
PWh: Petawatt hour
kg: Kilogram
GJ^−1^: Per Gigajoule
ppm: Parts per million
Pg: Petagram
